# East Asian *Cryphalus* Erichson (Curculionidae, Scolytinae): new species, new synonymy and redescriptions of species

**DOI:** 10.3897/zookeys.995.55981

**Published:** 2020-11-18

**Authors:** Andrew J. Johnson, You Li, Michail Yu. Mandelshtam, Sangwook Park, Ching-Shan Lin, Lei Gao, Jiri Hulcr

**Affiliations:** 1 School of Forest Resources and Conservation, University of Florida, Gainesville, FL, 32611, USA; 2 Department of Forest Protection, Wood Science and Game Management, Saint-Petersburg State Forest Technical University named after S.M. Kirov, Institutskii per., 5; 194021, Saint-Petersburg, Russia; 3 Research Institute of Forest Insect Diversity, Namyangju, 12113, South Korea; 4 Department of Entomology, National Taiwan University, Taipei, Taiwan; 5 Shanghai Academy of Landscape Architecture Science and Planning, Key Laboratory of National Forestry and Grassland Administration on Ecological Landscaping of Challenging Urban Sites, Shanghai 200232, China; 6 Department of Entomology, University of Florida, Gainesville, FL, 32611, USA

**Keywords:** Bark beetle, broadleaf, fruit tree, *
Hypocryphalus
*, mango, mulberry, new species, new taxa, pest, pseudo-cryptic species

## Abstract

*Cryphalus* Erichson, 1836 is a taxonomically challenging genus. It is particularly speciose in Asia. Many species are minor pests of fruit tree crops and forest products. We review collections from East Asia, using external morphology, internal morphology and genetic markers with a focus on sub-tropical species from fruit trees. Four new species are described; *Cryphalus gnetivorus* Johnson, **sp. nov.**, *C. itinerans* Johnson, **sp. nov.**, *C. morivorus* Johnson, **sp. nov.**, and *C. paramangiferae* Johnson, **sp. nov.** Ten species are redescribed to enable accurate identification: *C. artocarpus* (Schedl, 1939), *C. dilutus* Eichhoff, 1878, *C. dorsalis* (Motschulsky, 1866), *C. exiguus* Blandford, 1894, *C. kyotoensis* Nobuchi, 1966, *C. lipingensis* Tsai & Li, 1963 (= *C. kesiyae* Browne, 1975, **syn. nov.**), *C. mangiferae* Stebbing, 1914 (= *C. artestriatus* Browne, 1970, **syn. nov**.), *C. meridionalis* (Nobuchi, 1975), *C. scopiger* Berger, 1917, and *C. viburni* Stark, 1936. Additional records from new localities and new hosts are also presented.

## Introduction

Global forest health is threatened by a global redistribution of species introducing novel pathogens or vectors, coupled with stress from land management and climate change. Understanding the current biodiversity is a critical step in recording, reporting, and mitigating introductions of pests which affect the health of forest and agricultural systems. Bark and ambrosia beetles (Curculionidae: Scolytinae) are key drivers of change because of their roles in tree mortality, either via attacks overwhelming plant defences, or as vectors of disease (Six 2020). While a few bark beetle groups receive substantial attention of researchers, many are neglected. This has resulted in lack of revisionary systematics, difficulties in identification and taxonomic and nomenclatural complexity in most bark beetle groups.

*Cryphalus* Erichson, 1836 contains 252 known species of minute beetles ranging from 0.8 mm to 3.0 mm. Most of their diversity occurs in Asia, Australasia, and Oceania, with fewer species known from Africa and Europe, and a small number of species occurring in the Americas. Additionally, five putative species are introduced to the Americas (including *C. brasiliensis* Schedl, 1976 and *C. robustus* Eichhoff, 1872, but are only known from types and may represent extinct ephemeral populations).

*Cryphalus* typically live on recently dead or dying plant tissue under bark, but a few species are known to attack weakened (but still living) parts of trees and/or can vector plant pathogens (Jankowiak and Kolařík 2010). Known examples include *Ceratocystis* diseases of mango in the Middle East (Iqbal and Saeed 2012), and *Cryphalus* sp. attacking *Calophyllum* spp., vectoring *Verticillium calophylli* (Wiehe) W. Gams in the Seychelles (Wainhouse et al. 1998). *Cryphalus* have also been associated with the decline of other commodity trees such as figs (*Ficus carica* L.) (Faccoli et al. 2016), mulberry (*Morus* spp.) (Luo 2002) and loquat (*Eriobotrya japonica* (Thunb.) Lindl.) (Zheng et al. 2019), but without investigation to the role of pathogens.

Identification of *Cryphalus* is difficult, and they are often left without species identities (e.g., Zhao et al. 2004; Masuya et al. 2007; Hu et al. 2019), excluded in biodiversity surveys (e.g., Hulcr et al. 2007) and taxonomic work, or described with limited material and inadequate descriptions for diagnosis (e.g., Schedl 1942). Even economically important species, such as those on mango, have widespread identification errors which span decades (Johnson et al. 2017).

The beetles are challenging to identify due to their small size and soft bodies. Museum specimens are often discoloured and imploded, require high magnification microscopes to clearly see the diagnostic characters, or require dissection of the proventriculus or aedeagus, for which most species do not have adequate references to enable identification.

East Asia has a long history of taxonomic work on *Cryphalus*, from the meticulous work by Pang-hwa Tsai, Chao-lin Li, Huifen Yin, and Akira Nobuchi (e.g., Tsai and Li 1963; Nobuchi 1975; Yin et al. 1984). However, the subject of most of these studies were the species attacking coniferous forests, with very minimal treatment of species from broadleaf hosts. Taxonomic confusion with the now synonymised *Hypocryphalus* Hopkins, 1915 also contributed to the lack of treatment of species from broadleaf hosts- species previously classified in this genus, despite being morphologically very similar to certain species within the genus *Cryphalus*.

Developments in molecular tools give promise to enable accurate and rapid identification of difficult taxa, but rely on studies linking the identification of vouchered material, thorough descriptions, or sequence data with vouchered representatives (Cognato et al. 2020). The focus of this study is to provide data to enable accurate detection and study of *Cryphalus* species breeding in angiosperm trees in East Asia. Here, four species are described, ten species are redescribed, two names are synonymised, notes on the taxonomic history and new records are given for several East Asian species.

## Materials and methods

Specimens were obtained from various collection methods, including hand collecting from plant material and traps by the authors.

Specimens were initially sorted and identified with a stereo microscope (Olympus SZX16). Photographs were taken with taken with a digital SLR (Canon rebel t3i) mounted on an Olympus UIS2 system (BX53 microscope) with 5× – 40× objectives, illuminated by diffused halogen lights. Photographs were stacked with Helicon Focus (Helicon Soft) using the pyramid stacking algorithm (method:C), and edited in Photoshop (version CC2015, adobe.com). The edits were limited to correcting colour and removal of background objects.

Material was studied in ethanol, partially dried for photography, and later side-mounted on card points using Gelva ethanol soluble PVA (no longer manufactured). Pieces of dissected specimens were stored in ethanol or used for attempted DNA extraction.

Specimen data are maintained with unique identifiers in the UFFE collection database, which may refer to specimens owned and/or physically housed elsewhere. A UFFE id refers to one or a group of specimens housed in the same place sharing collection and identification information. If known, other repository identifiers are provided for specimens from other collections. Format of the material examined includes with records separated by a bullet point, and localities arranged by their current name, from larger administrative regions to smaller. Historic locations, where misinterpretations of localities are possible, have the label data quoted verbatim in addition to the locality. The following systems were used: Revised Romanization of Korean; Hanyu Pinyin (ISO 7098, Chinese); BGN/PCGN Romanization of Russian.

Taxonomic work follows the most recent review and reclassification treating *Cryphalus* Erichson (Johnson et al. 2020), which contains the full references and quotations of species treated and their synonyms. Holotypes were deposited in a state-supported taxonomic institution within each respective originating country, and paratypes are distributed to various international collections, particularly those in Asia where they would be useful to identify locally collected specimens. Types of existing specimens were examined directly or from photographs. Type material examined was clearly labelled as such and compared to the original description.

The following acronyms are used for specimens.

**FSCA**USA, Florida, Gainesville, Florida State Collection of Arthropods.

**IOZ**China, Beijing, Chinese Academy of Sciences, National Zoological Museum of China, Institute of Zoology.

**MZB**Indonesia, LIPI Research Center of Biology, Division of Zoology, Museum Zoologicum Bogoriense, Widyasatwaloka, Cibinong.

**NHMUK**United Kingdom, London, The Natural History Museum.

**NIAES** Japan, Ibaraki, Tsukuba, National Institute of Agro-Environmental Sciences (ITLJ).

**NMNS** Taiwan, Taichung, National Museum of Natural Science.

**RIFID** South Korea, Namyangju, Research Institute of Forest Insect Diversity.

**UFFE**USA, Florida, Gainesville, University of Florida Forest Entomology Collection.

**USNM**USA, Washington D.C., National Museum of Natural History.

**ZIN**Russia, St. Petersburg, Russian Academy of Sciences, Zoological Institute.

*Cryphalus* are very diverse and many undetermined specimens were observed. To avoid description of aberrant morphotypes or names which cannot be reliably identified in the future, new species were only described based on large series (at least 20 mature individuals) from multiple locations and for samples for which a DNA sequence had been obtained. Additional descriptions were provided for some species with economic significance.

Species are primarily described with a morphological species concept. To be considered a species, individuals have a distinct set of morphological characters based on a reasonable sample which the authors assume represents an evolutionary unit. Taxonomic changes are registered with ZooBank (Polaszek et al. 2005). For species of likely economic importance, a vernacular name is suggested in Chinese with an English translation. Chinese scientific literature uses vernacular names widely but sometimes inconsistently (Chen et al. 2017), so a name is suggested to promote consistency of use and prevent the establishment of non-informative or misleading names.

DNA sequence data were obtained when possible, to facilitate future molecular identification and corroborate species delimitation. The method used are as used by Johnson et al. (2017). Briefly, DNA was extracted from specimens using Qiagen DNeasy extraction kit (United States). DNA was amplified and sequenced with primers for 28 S (28S_A4285R: CCTGACTTCGTCCTGACCAGGC, 28S_S3690F: GAGAGTTMAASAGTACGTGAAAC; Sequeira et al. 2000) and COI (COI-LepF1: ATTCAACCAATCATAAAGATATTGG, COI-LepR1: TAAACTTCTGGATGTCCAAAAAATCA; Hebert et al. 2004). An approximate phylogeny was estimated using an alignment of 28S sequences listed in Table 1, inferred with MrBayes (version 3.2.7, Ronquist et al. 2012) using default settings.

Morphological terminology follows contemporary literature for bark and ambrosia beetles. Particularly, the funiculus includes the pedicel. Serrations are the asperities along the anterior margin of the pronotum. Converging aciculations refers to the texture appearing scratched, with irregular grooves and ridges, typically on the frons of some *Cryphalus* spp. converging on the epistoma. Lengths and proportions are given assuming a natural positioning of the beetle and given when viewed dorsally (e.g., pronotal length is from the apical margin to the base when viewed dorsally, not the diagonal distance). The pronotal profile refers to the shape when viewed dorsally, particularly where the pronotum is widest, which is usually at the base or in line with the summit. For the proventriculus, the closing teeth are the long teeth between the apical teeth and the masticatory brush.

The recorded host plants summarise the plant species from which the beetles were collected, primarily based on the material examined, with additional records from literature listed explicitly as uncertain. Similarly, the summarised distribution is based on examined material, listed as countries, with states or provinces listed specifically for the United States, Russia, and China. Relevant unconfirmed distribution records are explicitly cited as such.

An identification key is not provided at this time for the following reasons: all species in the region have not been thoroughly studied, especially the large diversity associated with coniferous trees, and the rate of species discovery is high and there are likely many more species yet to be reported, so keys may lead to misleading identifications. This study contributes towards a larger effort to make identification resources by providing thorough, complete descriptions and likely diagnostic characters with the intention to produce a key at a later date. Some types of species elsewhere are in poor condition and may correspond to aberrant individuals of the species included here but cannot be easily diagnosed. This approach is justified taxonomically, because it is important that names exist of the species in a timely manner. It is also justified economically, as several of the species here have been reported as pests, are related to pests, or are introduced elsewhere. Widely deposited type material, high resolution photographs and sequence data should ensure that conspecific specimens are identifiable in the future, enabling better descriptions of the distribution and biology of the species included, and enabling mitigation of potential pests.

## Results

### Phylogeny

**Figure 1.**
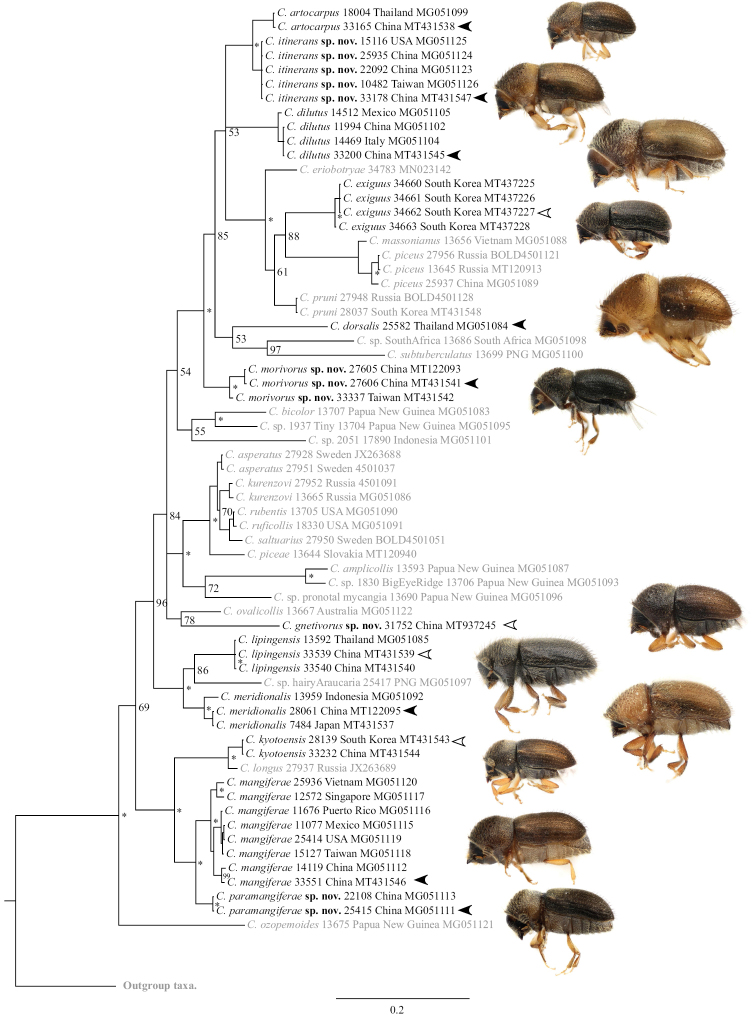
Preliminary phylogeny of *Cryphalus* spp. based on 28S, inferred using MrBayes using default parameters. Node labels indicate posterior probability (as %), 100% indicated with an asterisk. Outgroup taxa are listed in Table 1. Black text indicates treated species. Arrows correspond to the photographs, filled arrows indicate the exact specimen photographed, empty arrows indicate a specimen from the same collection being used.

The estimated phylogeny reveals some notable discoveries: Specimens from Thailand and China determined as *C. kesiyae* and *C. lipingensis* respectively are genetically identical, corroborating their morphological similarity (Figure 1). *Cryphalus exiguus* and another species from mulberry are distinct corroborating the morphological and distribution differences. *Cryphalus mangiferae* collected of *Choerospondias axillaris* (Roxb.) B.L. Burtt & A.W. Hill are genetically identical to specimens collected on *Mangifera indica* L. confirming *Choerospondias* as an alternative host.

### Systematics

#### Curculionidae Latreille, 1802

**Scolytinae Latreille, 1804**

**Cryphalini Lindemann, 1877**

##### Cryphalus

Taxon classificationAnimaliaColeopteraCurculionidae

Erichson, 1836

0912C8E2-B46B-5C14-9FB8-7C5C1A786442

###### Diagnosis.

Eye deeply emarginated; antennal club flat with three sutures marked by setae; third tarsal segment emarginated; the hypomeron with bifurcating setae (rare exceptions); proventriculus with large flat apical plates, tight median suture, often with sutural teeth, and distinct apical teeth, moustache-like, in multiple transverse layers; aedeagus with paired tegminal apodemes (few exceptions) (Johnson et al. 2020). Spiculum gastrale a simple, curved rod.

##### Cryphalus artocarpus

Taxon classificationAnimaliaColeopteraCurculionidae

(Schedl, 1939)

320DBD6C-7940-5C6F-9565-DCD8E5AFBF9A

**Figure 2.**
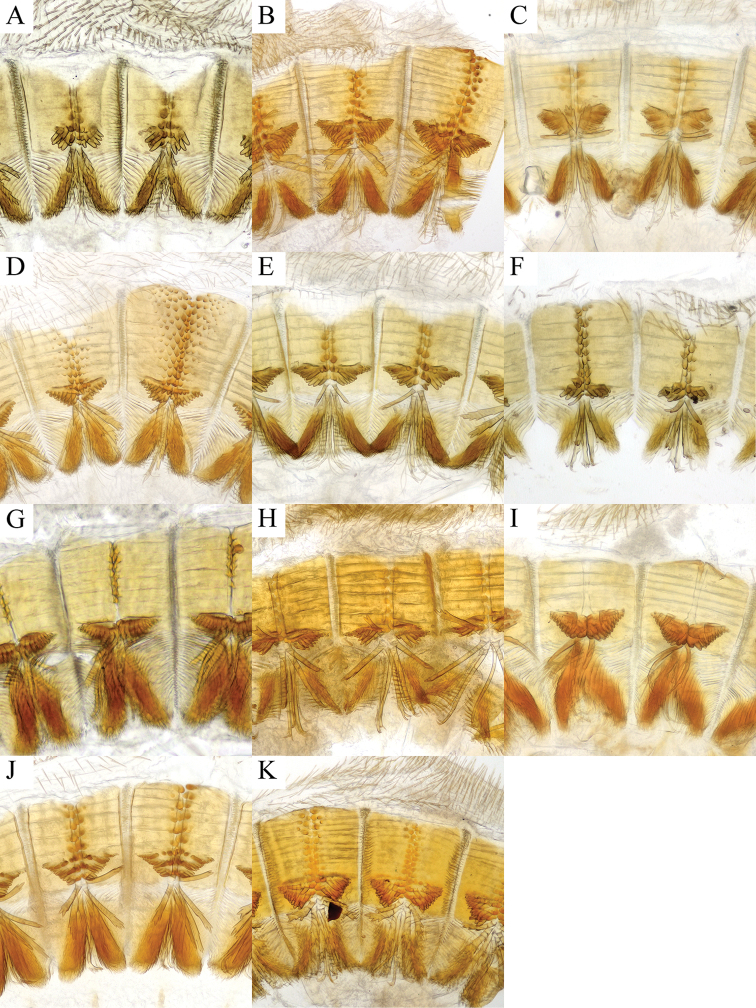
Proventriculus of **A***Cryphalus artocarpus*, UFFE:33165 **B***C. dilutus*, UFFE:34684 **C***C. dorsalis*, UFFE:25581 **D***C. gnetivorus*, UFFE:31753 **E***C. itinerans*, UFFE:33178 **F***C. kyotoensis*, UFFE:33232 **G***C. lipingensis*, UFFE:28048 **H***C. mangiferae*, UFFE:34966 **I***C. meridionalis*, UFFE:28061 **J***C. morivorus*, UFFE:27604 **K***C. paramangiferae*, UFFE:34965.

**Figure 3.**
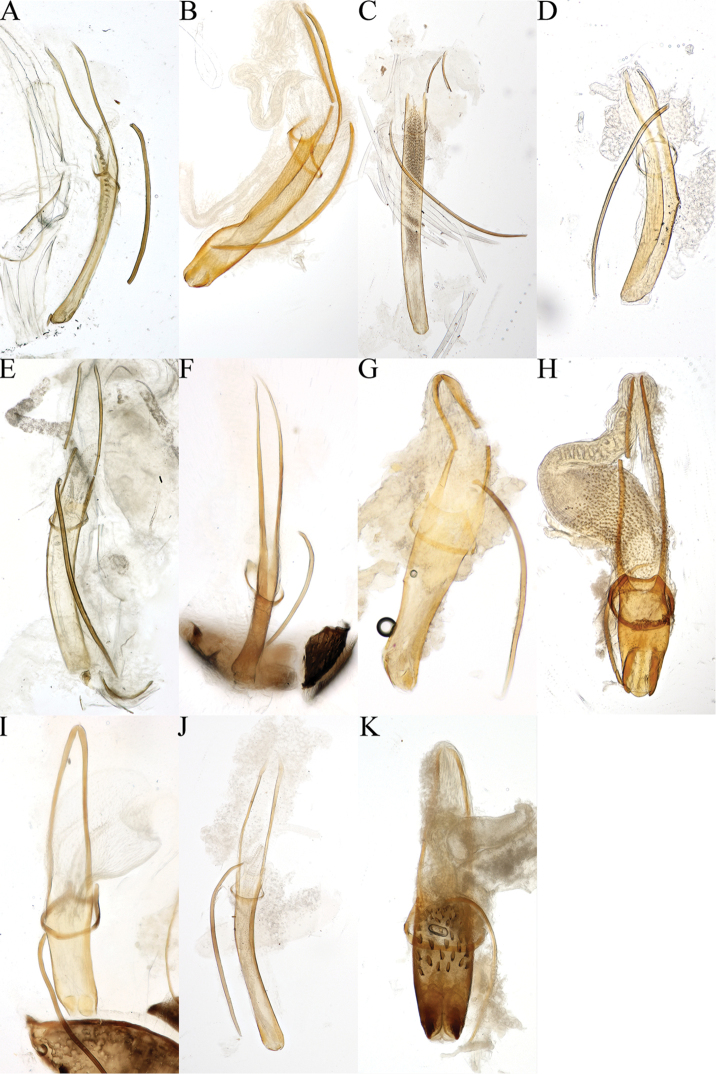
Aedeagus of **A***Cryphalus artocarpus*, UFFE:33165 **B***C. dilutus*, UFFE:34684 **C***C. dorsalis*, UFFE:25581 **D***C. gnetivorus*, UFFE:31753 **E***C. itinerans*, UFFE:33178 **F***C. kyotoensis*, UFFE:31745 **G***C. lipingensis*, UFFE:28048 **H***C. mangiferae*, UFFE:34966 **I***C. meridionalis*, UFFE:28061 **J***C. morivorus*, UFFE:27604 **K***C. paramangiferae*, UFFE:34965.

**Figure 4.**
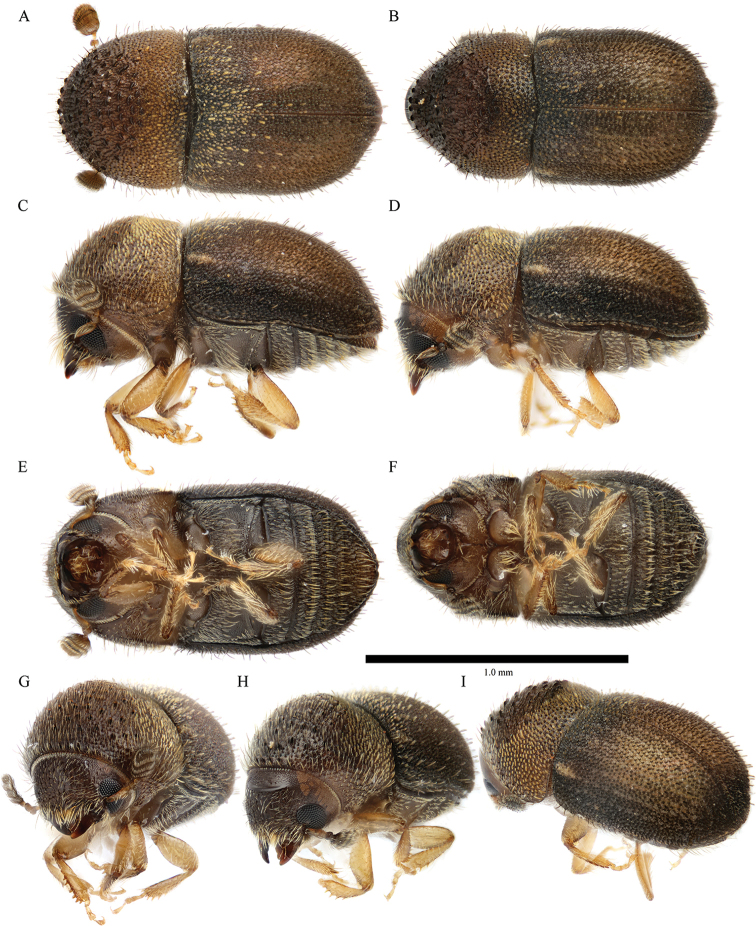
*Cryphalus artocarpus***A, C, E, G** female, UFFE:33166 **B, D, F** male, UFFE:33167 **H, I** male, UFFE:33165.

Figures 2A , 3A , 4A–I

Ericryphalus artocarpus Schedl, 1939: 432 (Malaysia).
Cryphalus artocarpus Schedl, 1958: 498 (Malaysia).
Cryphalus brownei Wood, 1992: 432 (unnecessary replacement name).

###### Type material examined.

Malaysia • 1 ♀ ***Holotype****Cryphalus artocarpus* Schedl, 1958; Sarawak, Siburan, Semenggoh; 23 Aug. 1957; F. G. Browne leg.; ex. *Artocarpus*; on branch of *Artocarpus*; 5833; UFFE: 26193; (NHMUK).

###### Other material examined.

China • 4 ♀♀, 4 ♂♂; Hainan; 儋州市宝岛新村试验场七队桑园 [Danzhou, Baodaoxincun, No.7 farm, Mulberry field]; 19.51°N, 109.49°E; 14 Mar. 2019; Fuping Lu, and Shengchang Lai leg.; ex. *Morus*; “; samples degraded.; UFFE:33536; (UFFE) • 2 ♀♀, 1 ♂; Yunnan, Xishuangbanna, Sanchahe Nature Reserve; 22.1631°N, 100.8709°E; 30 May 2008; Anthony I. Cognato leg.; ex. *Ficus*; vial 133; Sanchahe Nature Reserve; collect from host tree phloem; 30/May/2008; Cognato coll; UFFE:11411; (UFFE) • 1 ♀; Yunnan, Xishuangbanna, Xishuangbanna Tropical Botanical Garden; 21.92°N, 101.27°E; 12 Jul. 2014; Craig Bateman leg.; EtOH trap; moved from vial 7778; UFFE:33166; (UFFE) • 1 ♂; same collection data; UFFE:33167; (UFFE) • 1 ♂; same collection data; DNA: 28S:MT431538; UFFE:33165; (UFFE) • 12 ♂♂, 12 ♀♀; Yunnan, Xishuangbanna, Xishuangbanna Tropical Botanical Garden; 21.92°N, 101.27°E; 12 Jul. 2014; Craig Bateman leg.; EtOH bottle trap; UFFE:14088; (NHMUK, 1♀, 1♂; FSCA, 1♀, 1♂; MZB, 1♀, 1♂; NIAES, 1♀, 1♂;NMNS, 1♀, 1♂; IOZ, 1♀ IOZ(E)2057936, 1♂ IOZ(E)2057937; RIFID, 1♀, 1♂; UFFE, 2♀♀; USNM, 1♀, 1♂; ZIN, 1♀, 1♂).

Thailand • 1 ♂; Phatthalung, Srinagarindra District, Khao Banthat wildlife sanctuary; Jun. 2015; S. Steininger, and W. Sittichaya leg.; DNA: 28S:MG051099; UFFE:18004; (UFFE)

###### Diagnosis.

This species can be diagnosed from similar *Cryphalus* in East Asia by the small size (1.10–1.30 mm), by the large pronotal disc one third of the length of the pronotum, by the stout elytra with a finely tuberculate surface texture, and by the male protibiae with similar setae to the females.

**Female.** Length 1.10–1.30 mm (holotype 1.25 mm). Proportions 2.1× as long as wide. Frons with minute aciculations, barely visible, converging to the epistoma. Antennal club with three weakly procurved sutures marked by coarse and long setae. Antennal funiculus with four segments, length shorter than the scape. Gular surface with evenly spaced hair-like setae. Protibiae and protarsi with only straight, hair-like setae. Pronotal colour dark brown on slope, sometimes a lighter brown on disc. Pronotal profile broadly rounded, slightly wider in line with summit. Pronotal margin armed with six to ten serrations, separated by approximately their width, the outer one or two pairs smaller. Pronotal declivity with more than 40 asperities (holotype has 51). Pronotal disc approximately one third the length of the pronotum, gently sloped, weakly tuberculate surface texture (obscured by scale-like setae). Pronotal vestiture on anterior and lateral slope hair-like. Pronotal vestiture on disc and postero-lateral regions mixture of scale-like and paddle-like, the scale-like setae 1× long as wide with a tridentate tip. Suture between pronotum and elytra weakly sinuate. Scutellum very small, barely visible. Elytra 1.5× as long as pronotum, brown to translucent yellow-brown, darker at base, broadly rounded with no clear elytral disc or a transition to the declivity, slightly angulate on lateral regions of the declivity on interstriae 5. Elytral surface densely punctures with small tubercles, especially on the basal half. Striae barely visible as rows of punctures and hair-like setae. Interstrial bristles erect, flattened with rounded tips, shorter towards the elytral suture. Interstrial ground vestiture tridentate, approximately one to two × as long as wide, translucent brown with a weak iridescence, sometimes light brown near the base of the elytra. Protibiae and protarsi with only straight, hair-like setae. Mesocoxae moderately separated, more than distance between metacoxae. Proventriculus not examined.

**Male.** Similar to female except: Length 1.05–1.20 mm. Frons weakly aciculate with a glossy transverse carina above upper level of eyes. Gular surface shining around suture, surrounded by sparse setae. Pronotal profile widest in line with summit. Pronotal profile anterior to summit triangular. Pronotal margin with four to six marginal asperities, spaced approximately by twice their width. Pronotal declivity with almost straight slope, fewer and smaller asperities than the female. Pronotal disc with strongly tuberculate surface obscured by scale-like setae. Elytral surface densely punctured and strongly tuberculate, especially on basal half. Protibiae and protarsi with hair-like setae which are curved, slightly larger than on female. Last abdominal ventrite clearly emarginated. Proventriculus sutural teeth rounded, in two overlapping longitudinal rows. Apical teeth extend laterally to less than two thirds of the plate. Closing teeth short, mix of palmate shorter teeth and longer, tapered, branched teeth extending beyond masticatory brush. Masticatory brush slightly less than half of the proventricular length. Aedeagus long, weakly sclerotised. Penis apodemes approximately two thirds as long as penis body. Tegmen with two ventral apodemes, which are longer than distance between them. End plates indistinct, barely sclerotised.

###### Distribution.

China (Yunnan, Hainan); Thailand; Malaysia.

###### Recorded plant hosts.

Moraceae: *Artocarpus* sp., *Artocarpus elasticus* Reinw. ex Blume., *Ficus* sp., *Morus* sp.

###### Remarks.

Abundant in ethanol bottle trap samples in Yunnan. This species was listed as “spThailandScaly” in Johnson et al. (2017).

##### Cryphalus dilutus

Taxon classificationAnimaliaColeopteraCurculionidae

Eichhoff, 1878a

9ABF5ECB-A4DB-5D1E-B24A-981D1F66A53A

**Figure 5.**
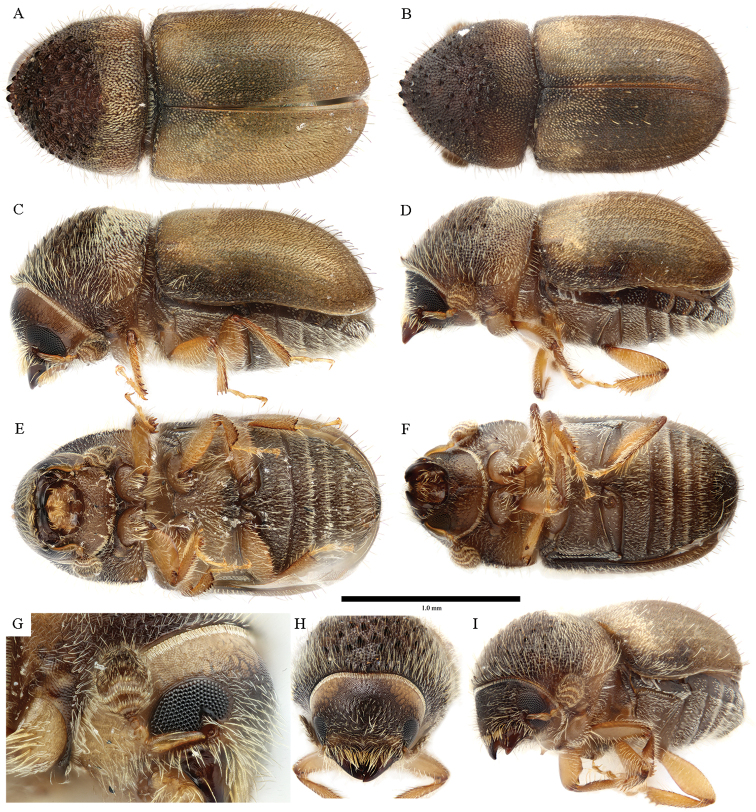
*Cryphalus dilutus***A, C, E, G, H** female, UFFE:33200 **B, D, F, I** male, UFFE:34684.

Figures 2B , 3B , 5A–I

Cryphalus dilutus Eichhoff, 1878a: 384 (Myanmar); Eichhoff, 1878b: 490 (Myanmar).

###### Type material examined.

Myanmar • 1 ♂ ***Holotype***; “Hindostan”; UFFE:14961; (NHMW).

###### Other material examined.

China • 1 ♀; Guangdong, Shenzhen, Yantian; 22.5889°N, 114.2842°E; 08 Apr. 2017; Wei Lin leg.; dissected; moved from vial 17740; DNA: 28S:MT431545, COI:MT431649; UFFE:33200 • 1 ♀; Guangdong, Shenzhen, Yantian; 22.5889°N, 114.2842°E; 08 Apr. 2017; Wei Lin leg.; UFFE:33240; (UFFE) • 1 ♂; 深圳水库 [Shenzhen Reservoir]; 22.5945°N, 114.1797°E; 11 Jul. 2018; 阮用颖 [Yongying Ruan leg.]; light trap; UFFE:34064; (UFFE) • 1 ♀, 1 ♂; Guangdong, Zhuhai, Shixi Park; 22.2847°N, 113.5539°E; 01 Aug. 2018; Wei Lin, and You Li leg.; UFFE:34963 (IOZ, 1♀ IOZ(E)2057934, 1♂ IOZ(E)2057935) • 1 ♂; same collection data; dissected; UFFE:34684 (UFFE) • 1 ♂; specific origin unknown; 17 Dec. 2013; ex. *Ficus*; Interception at USA, Oberlin, from shipment of *Ficus* sp.; DNA: 28S:MG051102, COI:MG051150; UFFE:11994; (UFFE) • 1 ♀; Yunnan, Jinghong, Yunnan Institute of Tropical Crops; 22°N, 100.78°E; 13 Mar. 2019; Quan Zhou, and Shengchang Lai leg.; 20190313008 trap; UFFE:34041; (UFFE).

Mexico • 1 ♀; Tabasco, La Frontera; 18.6138°N, -92.5749°E; 19 Aug. 2014; Thomas H. Atkinson, and S. Burgos leg.; ex. *Mangifera indica*; shaded-out branches, 3–5 cm. diameter; UFFE:14892; (UFFE) • 1 ♂; same collection data; UFFE:14893; (UFFE).

Oman • 1 ♀; Mar. 2005; Randy Ploetz leg.; ex. *Mangifera indica*; labelled: “Oman: III 2005; Randy Ploetz; Ex. Mangifera indica”; UFFE:12479; (FSCA).

###### Diagnosis.

This species can be diagnosed by the combination of the transverse carina on the male frons, by the pronotal margin which projects slightly, by the scale-like setae on the pronotal disc, by the barely apparent striae, and by the long spatula-shaped setae on the protibia and the spur on the mesofemur of males. The spine on the mesofemur is known exclusively in this species among all Scolytinae.

**Female.** Length 1.50–2.20 mm. Proportions 2.05× as long as wide. Frons simple, convex, with sparse but evenly distributed setae pointing towards its centre. Antennal club with three sutures on the basal half marked by coarse and long setae, the first weakly procurved, second and third more strongly procurved, and a weakly visible procurved line of coarse setae in the upper (distal) half. Antennal funiculus with four or five segments, the pedicel is slightly shorter than the other segments combined. Gular surface with evenly spaced hair-like setae. Pronotal colour brown, typically similar to head and elytra. Pronotal profile slightly triangular, widest in line with centre of pronotal disc. Pronotal margin rounded but protruding downward slightly, armed with four to eight serrations, the median pair distinctly larger, contiguous or separated by approximately half of their width, the outer pairs smaller separated by more than their width. Pronotal declivity with approximately 60 asperities. Pronotal disc approximately one third the length of the pronotum, gently sloped, with surface texture rugose/closely punctured, partially obscured by scale-like setae. Pronotal vestiture hair-like and dark coloured on anterior and lateral slope, and a mixture of blonde scale-like and bristle-like, on disc and postero-lateral regions, with the scale-like setae 1–2× long as wide with a tridentate tip. Suture between pronotum and elytra weakly sinuate. Scutellum very small, barely visible when elytra closed. Elytra 1.6× as long as pronotum, brown to translucent yellow-brown, broadly rounded with no clear elytral disc or a transition to the declivity. Striae barely visible as rows of punctures and hair-like setae. Interstrial bristles erect, coarse bristles, shorter towards the elytral suture, evenly distributed across elytra. Interstrial ground vestiture tridentate, approximately 1–2× as long as wide, translucent brown with a weak iridescence, except the basal/anterior third of elytra which is blonde. Antero-lateral margin of elytra with some blonde hair-like setae, more dense than the interstrial bristles over the rest of the elytra. Protibiae and protarsi with only straight, hair-like setae. Mesocoxae moderately separated, more than distance between metacoxae. Mesofemur with a slightly raised patch in the centre of the ventral face. Proventriculus sutural teeth of irregular size, confused, in two or more longitudinal rows. Apical teeth extend laterally over the entire segment. Closing teeth weakly branched, tapered, extending beyond masticatory brush. Masticatory brush with fine teeth, short, less than half the proventricular length.

**Male.** Similar to female except: Length 1.50–2.20 mm (type is 1.67 mm). Frons with straight transverse carina and sulcus above the level of eyes. Pronotal profile triangular, protruding apically. Pronotal declivity almost flat (not broadly rounded). Protibiae and protarsi with large spatula-shaped setae. Mesofemur with a distinct spine in the centre of the ventral surface. Last abdominal ventrite clearly emarginated. Proventriculus same as female. Aedeagus long. Penis apodemes shorter than penis body. Tegmen with paired apodemes about as long as distance between them. End plates sclerotised.

###### Distribution.

China (Guangdong, Yunnan); Myanmar; also recorded from Malta; Italy; Tunisia; UAE; Oman; India; Pakistan; Bangladesh; Mexico (all Johnson et al. 2017).

###### Recorded plant hosts.

Anacardiaceae: *Mangifera indica* L.; Moraceae: *Ficus carica* L., *F. retusa* L., *F. microcarpa* L.f., *Ficus bengalensis* L. (no new material from plant hosts, all cited in Johnson et al. 2017).

###### Suggested vernacular name.

Chinese: 刺足梢小蠹 [= spur-footed twig bark beetle]; English: Spurred bark beetle.

###### Remarks.

This species is associated with diseases of mango and die-off of edible fig. These new records confirm that it is widespread in Southern China; previously it was only known from an interception.

##### Cryphalus dorsalis

Taxon classificationAnimaliaColeopteraCurculionidae

(Motschulsky, 1866)

13649E89-ADF9-5E6B-AC0D-7F282E54A6B9

**Figure 6.**
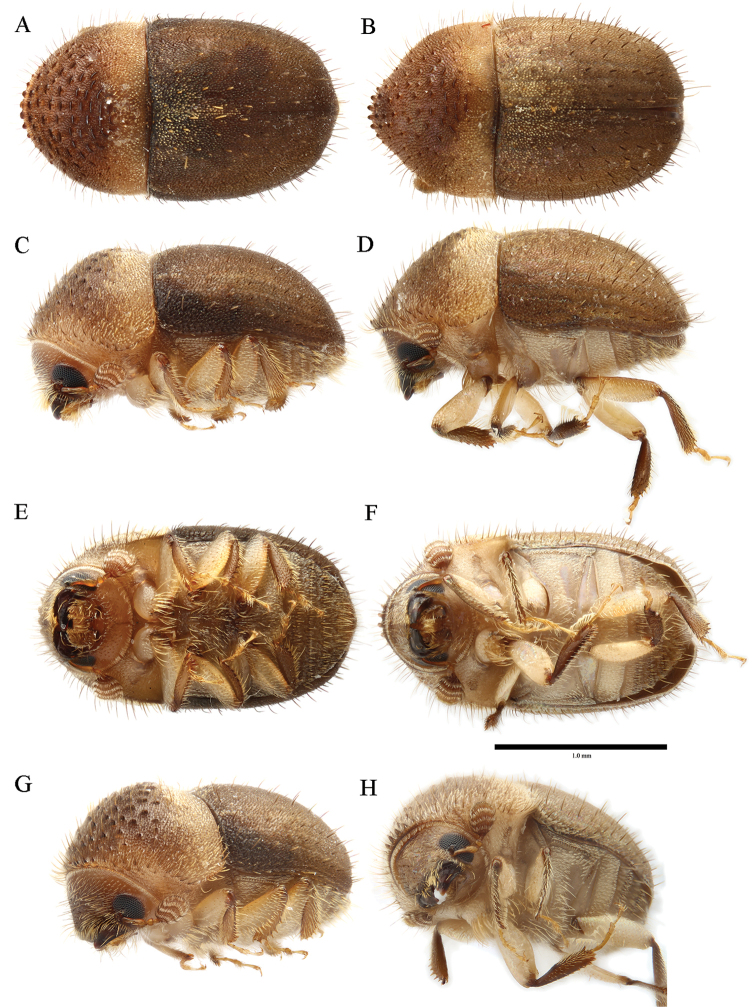
*Cryphalus dorsalis***A, C, E, G** female, UFFE:34070 **B, D, F, H** male, UFFE:21476.

Figures 2C , 3C , 6A–H

Hypoborus dorsalis Motschulsky, 1866: 403 (India).
Hylesinus sericeus Motschulsky, 1866: 402 (Sri Lanka).
Hypoborus nebulosus Motschulsky, 1866: 403 (India).
Cryphalus indicus Eichhoff, 1878a: 384 (Myanamar).
Cryphalus indicus Eichhoff, 1878b: 489 (Myanamar).

###### Other material examined.

China • 1 ♀; Hainan, Qiongzhong, Wanling; 19.2115°N, 109.9548°E; 26 Oct. 2016; You Li leg.; Light trap with ethanol; UFFE:34070; (UFFE) • 1 ♂; Yunnan, Xishuangbanna, Xishuangbanna Tropical Botanical Garden; 27 Jul. 2014; Craig Bateman leg.; EtOH live trap; specimens with crystalline structure, degraded; UFFE:21476; (UFFE).

Thailand • 1 ♂; Phatthalung, Srinagarindra District, Khao Banthat wildlife sanctuary; Jun. 2015; S. Steininger, and W. Sittichaya leg.; UFFE:25581; (UFFE) • 1 ♀; same collection data; DNA: 28S:MG051084; UFFE:25582; (UFFE).

Vietnam • 1 ♀; Hải Châu District, Da Nang; 10 Dec. 1966; H. P. Schurtleff leg.; S.L.Wood Collection; UFFE:12188; (USNM).

###### Diagnosis.

The combination of the size (1.60–1.90 mm), body proportions (1.75 × as long as wide), the transverse ridge on the male frons, scale-like setae on the pronotal disc, the very short pronotal disc, the smooth elytra with barely visible rows of strial punctures, and the presence of sparse interstrial bristles on only odd-numbered interstriae, and long spatula-shaped setae on the male protibiae distinguish this species from others in East Asia.

**Female.** Length 1.60–1.90 mm. Proportions 1.75× as long as wide. Frons simple, convex, with sparse, evenly distributed erect setae. Antennal club with three procurved sutures marked by coarse and long setae, the most distal slightly more procurved. Antennal funiculus with four segments, the pedicel is slightly shorter than the other segments combined. Gular surface with evenly spaced hair-like setae. Pronotal colour brown, lighter on apical third. Pronotal profile slightly triangular, widest in line with centre of pronotal disc, 0.65× as long as wide. Pronotal margin rounded, armed with four to eight serrations, the median pair slightly larger, contiguous, or separated by approximately half of their width, the outer pairs smaller separated by more than their width. Pronotal declivity with approximately 50 asperities, otherwise smooth. Pronotal disc approximately one quarter the length of the pronotum, gently sloped, with surface texture weakly tuberculate. Pronotal vestiture hair-like and dark coloured on anterior portion pointing towards summit, and scale-like setae over pronotal disc. Suture between pronotum and elytra weakly sinuate. Scutellum very small, barely visible when elytra closed. Elytra 1.6× as long as pronotum, brown to translucent yellow-brown, elytral disc short, less than 1/3 length of elytra, and broadly rounded. Striae weakly visible as rows of punctures and hair like setae. Interstrial bristles erect, coarse bristles, shorter with a rounded tip on apical half, becoming longer and pointed on the declivity and lateral regions, only present on interstriae 1, 3, and 5 on declivity. Interstrial ground vestiture tridentate, approximately 2× as long as wide, translucent brown with a weak iridescence, except a few on the basal area near scutellum, which are blonde, antero-lateral margin with hair-like setae shorter than interstrial bristles. Procoxae with coarse, hair-like setae. Protibiae and protarsi with only straight, hair-like setae. Mesocoxae moderately separated, more than distance between metacoxae. Proventriculus sutural teeth in multiple irregular rows. Apical teeth extend two thirds the width of the segment. Closing teeth longer than masticatory brush, branched near tips. Masticatory brush with fine teeth, short, less than half the segment length.

###### Male.

Similar to female except: Length 1.60–1.80 mm. Frons with straight transverse carina and sulcus above the level of eyes. Gular surface impressed and glabrous, surrounded by sparse hair-like setae. Pronotal profile triangular, almost constricted on antero-lateral edges. Pronotal declivity almost flat (not broadly rounded). Procoxae with large feather-like setae. Protibiae and protarsi with large spatula-shaped setae along inner margin. Last abdominal ventrite emarginated. Proventriculus same as female. Aedeagus not examined.

###### Distribution.

China (Hainan, Yunnan); Thailand; Vietnam.

###### Remarks.

Yin et al. (2002) listed *Cryphalus indicus* Stebbing, 1902, a primary homonym replaced by *Cryphalus strohmeyeri* Stebbing, 1914, as from Hainan. This record has subsequently been included in catalogues (Alonso-Zarazaga et al. 2017). Based on the descripted in Yin et al. (2002), the identity was probably intended as *Cryphalus indicus* Eichhoff, 1878a, a junior synonym of *C. dorsalis*. The description in Yin et al. (2002) clearly indicates the stout proportions (1.8× as long as wide) and the transverse ridge on the male frons, which correspond to *C. dorsalis* as we found in Hainan. Yin et al. (2002) listed the host as *Abies*, probably from other records or the original description, which does not grow in Hainan. Specimens of *C. strohmeyeri* were studied from elsewhere in China (USNM).

##### Cryphalus exiguus

Taxon classificationAnimaliaColeopteraCurculionidae

Blandford, 1894

DD73654D-FB35-52D7-BBA7-AE416100C202

Figure 7A–I

Cryphalus exiguus Blandford, 1894: 82 (Japan).

###### Type material examined.

Japan • 1 ♂ ***Holotype***; Fukushima, not recorded; 29 Jul. 1891; C. Lewis leg.; labelled “Japan // C. Lewis // 1910–320 ///// Fukushima // 26.VII-29.VII.91 // *Cryphalus exiguus* Bland. // NHMUK 010805962”; UFFE:26205; (NHMUK).

###### Other material examined.

Japan • 1 ♀; Yamagata, Tendo; 06 May 1982; I Ueno leg.; Photographed by SP; UFFE:28638; (NIAES).

Russia • 1 ♀; Sakhalin Oblast, South Kuril urban district, Kunashir Island, Tret’yakovo; 29 Jul. 2008; Michail Yu. Mandelshtam leg.; ex. *Morus australis*; small twigs, river valley; UFFE:31832; (FSCA).

South Korea • 1 ♂; Gangwon-do, Inje-gun, Inje-eub, Nambuk-ri; 38.0753°N, 128.1336°E; 23 Apr. 2013; Sangwook Park leg; UFFE:28122; (RIFID) • 1 ♀; same collection data; UFFE:28123; (RIFID) • 1; Gangwon-do, Inje-gun, Buk-myeon, Yongdae-ri; 38.2075°N, 128.3382°E; 08 May 2018; Sangwook Park leg.; specimen S3 by sequence only,; DNA: 28S:MT437225; UFFE:34660; (RIFID) • 1; Gangwon-do, Inje-gun, Buk-myeon, Hangye-ri; 38.1316°N, 128.3004°E; 08 May 2018; Sangwook Park leg.; specimen S5 sequence only; DNA: 28S:MT437226; UFFE:34661; (RIFID) • 1; Gangwon-do, Inje-gun, Buk-myeon, Hangye-ri; 38.1316°N, 128.3004°E; 08 May 2018; Sangwook Park leg.; specimen S6 by sequence only; DNA: 28S:MT437227; UFFE:34662; (RIFID) • 1; Gangwon-do, Pyeongchang-gun, Yongpyeong-myeon, Soksa-ri; 37.6673°N, 128.497°E; 20 May 2017; Sangwook Park leg.; specimen S11 sequence voucher; DNA: 28S:MT437228; UFFE:34663; (RIFID).

###### Diagnosis.

This species is diagnosed from others in East Asia by the size (1.30–1.55 mm), by the proportions (2.20× as long as wide), by the male frons with a short transverse carina, by the antennal sutures (approximately horizontal), by the setae on the pronotal disc (entirely hair-like), and by the elytral striae devoid of ground vestiture.

This species is very similar to *Cryphalus mandschuricus* Eggers, 1929, and differs by the transverse carina on the male frons (*C. exiguus*: narrower, not much more than two thirds of the width between eyes; *C. mandschuricus*: wider, almost as long as width between eyes), by the sutures on the antennal club (*C. exiguus*: almost straight, recurved at edges; *C. mandschuricus*: procurved, especially third suture), by the length of setae on the pronotal disc (*C. exiguus*: much longer than distance between setae; *C. mandschuricus*: barely as long as distance between setae), and by the length of the elytral disc of females (*C. exiguus*: about two thirds of the length of elytra, declivity steep; *C. manschuricus*: only half of elytral disc, declivity slope gradual). The aedeagus of *C. exiguus* was not examined, but that in *C. mandschuricus* has the tegmen apodemes much longer than the distance between them.

*Cryphalus morivorus*, described below, is similar and can be distinguished by the characters therein.

**Female.** Length 1.40–1.55 mm (holotype 1.50 mm). Proportions 2.20× as long as wide. Frons simple, convex, flat and shining up to the level of the eyes. Antennal club with three straight sutures marked by a mixture of coarse short setae and longer setae with length approximately the same as distance between sutures. Antennal funiculus with four funicular segments. Pronotal colour dark brown, similar to elytra and head. Pronotal profile widest at base. Pronotal margin armed with four or five serrations, irregularly spaced and sometimes partially fused. Pronotal declivity broadly rounded, with approximately 50 asperities. Pronotal disc occupies approx. one third of the length of the pronotum, gently sloped, uniformly weakly asperate. Pronotal vestiture of only gold, hair-like setae. Suture between pronotum and elytra very weakly v-shaped. Scutellum small, triangular. Elytra 1.85× as long as pronotum, dark brown, broadly rounded with no clear transition to the declivity. Elytral texture mostly smooth, with punctures from the ground vestiture much smaller than the diameter of the strial punctures, and interspaces smooth. Striae visible as rows of punctures, with no scale-like setae between strial punctures. Interstrial bristles erect, hair-like, slightly flattened with pointed tips, those near the elytral suture are shorter and broader than those in lateral regions. Interstrial ground vestiture tridentate, 1–2× as long as wide, with a weak iridescence. Protibiae and protarsi with only simple hair-like setae. Mesocoxae narrowly separated, only slightly more than metacoxae. Proventriculus not examined.

**Male.** Similar to female except: Length 1.30–1.50 mm. Frons with a smooth transverse carina above the level of the eyes. Pronotal declivity with asperities slightly more sparse and slope is straight rather than broadly curved. Protibiae and protarsi with coarse, long setae on the proximal face, near the apex. Last abdominal ventrite clearly emarginated. Proventriculus not examined. Aedeagus not examined.

###### Distribution.

Russia (Southern Kuriles: Kunashir and Shikotan Isles in Sakhalin Oblast); Japan; South Korea, North Korea (Ju, 1964, unconfirmed), China (North-East, Krivolutskaya, 1996, unconfirmed).

###### Recorded plant hosts.

Moraceae: *Morus australis* Poir., *M. alba* L.

###### Suggested vernacular name.

Chinese: 北桑梢小蠹 [Northern mulberry twig bark beetle]; English: Northern mulberry bark beetle; Korean: 뽕나무애나무좀; Russian: крифал шелковичный.

###### Remarks.

Frequently misspelled as “*exignus*”, with the first instance of this misspelling in Niisima (1909). The name *Cryphalus pilosus* Sasaki, 1899 has been treated as a junior synonym of *C. exiguus* (Wood and Bright 1992; Alonso-Zarazaga et al. 2017), based on the apparent synonymy by Niisima (1909). Upon inspection of the referenced material (Sasaki 1899), the synonymy is deemed to be a misinterpretation. The original publication, in Japanese, describes a pest of mulberry with the tentative identification as “Hylesinus (Xlechinus) pilosus?” [Sic], presumably referring to *Xylechinus pilosus* (Ratzeburg 1837) (Scolytinae, Hylurgini). There was no type material deposited or any indication that this was intended as a new species. Niisima’s remark was intended to correct the identification, rather than suggesting that the two species names are synonymous.

The original description (Blandford 1894) indicates that the pronotum contains scale-like setae (“…with a thin covering of scales and hairs”). This is not visible on the holotype and any other specimens seen from northern and central Japan. Crucially, this is the opposite of a character state which diagnoses this species (having only hair-like setae). We consider this an error in the species description rather than either damage or accidental replacement of the holotype. The weakly asperate texture of the pronotal disc can give the illusion of scales in non-diffuse lighting. Another diagnostic character for *C. exiguus* is the clearly visible striae devoid of ground vestiture, which is included in the original species description.

We did not see any specimens representing this species in China (see remarks for *C. morivorus*), but it is likely to be present in the north-eastern regions. The key by Tsai and Li (1963) clearly indicated prominent striae, suggesting specimens of *C. exiguus* were used when compiling the key and comparing to *C. manschuricus*.

**Figure 7.**
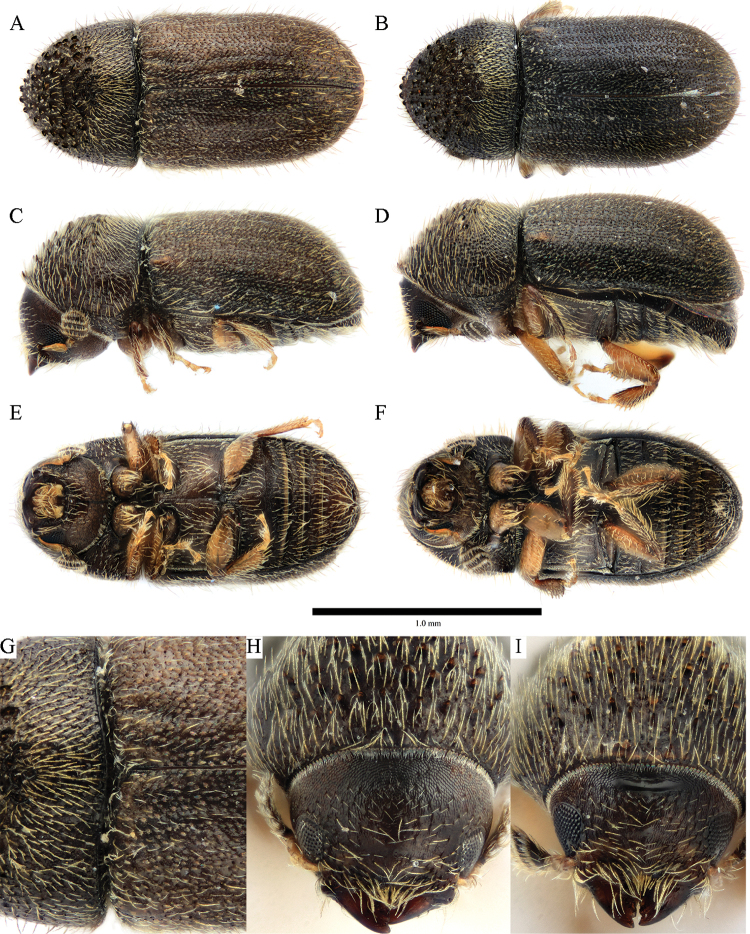
*Cryphalus exiguus***A, C, E, G** female, UFFE:34070 **B, D, F, H** male, UFFE:21476.

##### Cryphalus gnetivorus

Taxon classificationAnimaliaColeopteraCurculionidae

Johnson sp. nov.

336847BC-0ACE-59A1-ADF1-B06B10852B41

http://zoobank.org/320AFB38-5A27-458D-9A92-317274D8FC95

**Figure 8.**
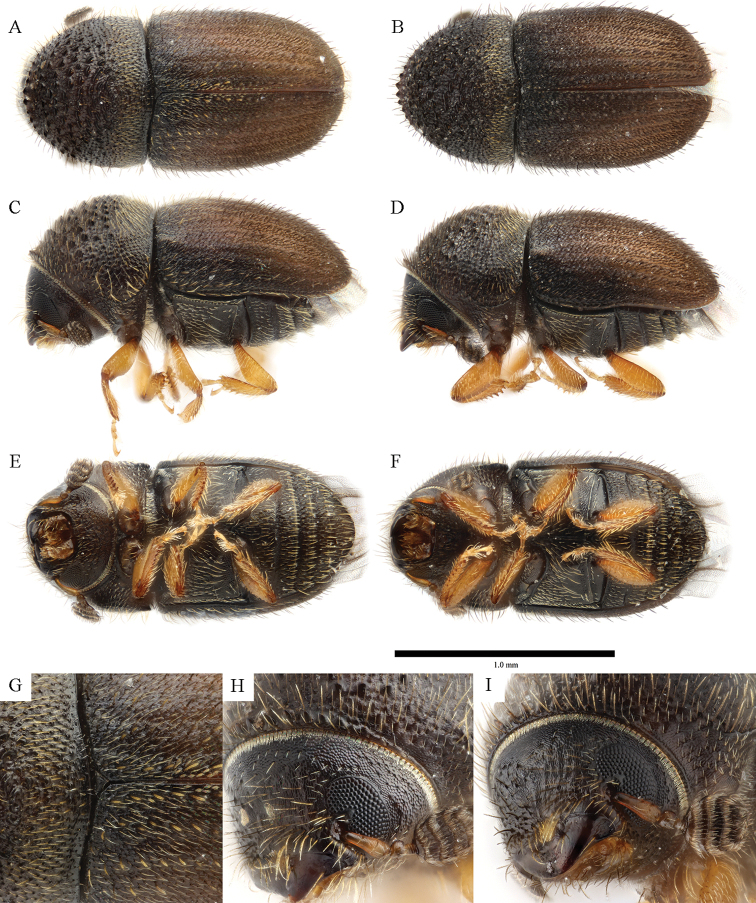
*Cryphalus gnetivorus***A, C, E, G, H** holotype, female, UFFE:31751 **B, D, F, I** paratype, male, UFFE:31753.

Figures 2D , 3D , 8A–I

###### Type material examined.

China • 1 ♀ ***Holotype***; Guangdong, Shenzhen, Dapeng dam; 22.6316°N, 114.4624°E; 12 Apr. 2018; You Li leg.; ex. *Gnetum luofuense* ; reared from dead vines; IOZ(E)225671; UFFE:31751 (IOZ) • 1 ♂ ***Paratype***; same collection data; dissected; UFFE:31753; (UFFE) • 1 ♂ ***Paratype***; same collection data; dissected; DNA: 28S:MT937245, COI:MT937225; UFFE:31752 (UFFE) • 1 ♂ ***Paratype***; same collection data; UFFE:31750 (UFFE) • 8 ♀♀, 7 ♂♂ ***Paratypes***; same collection data except 01 Jun. 2018; UFFE:34924 (NHMUK, 1♀, 1♂; FSCA, 1♀; MZB, 1♀, 1♂; NIAES, 1♀, 1♂; NMNS, 1♀, 1♂; IOZ, 1♂ IOZ(E)225672; RIFID, 1♀; USNM, 1♀, 1♂; ZIN, 1♀, 1♂).

###### Other material examined.

China • 1; Guangdong, Shenzhen, Yantian; 22.5889°N, 114.2842°E; 08 Apr. 2017; Wei Lin leg.; UFFE:33241.

###### Type locality.

China, Guangdong, Shenzhen, Dapeng dam (22.6316°N, 114.4624°E).

###### Diagnosis.

*Cryphalus gnetivorus* can be distinguished from most of the East Asian *Cryphalus* by the combination of the size (1.20–1.50 mm), the proportions (twice as long as wide), by the male frons with minute transverse aciculations in upper median area, by the short elytral disc and long, gradual declivity, by the interstrial bristles of approximately even length, widened and rounded at tips, pointing posteriorly, by the female interstrial ground vestiture which is hair like near base and tridentate scale-like on declivity, and by the proventriculus with a wide area of sutural teeth, occupying half of the segment width.

**Female.** Length 1.25–1.50 mm (holotype 1.45 mm). Proportions 2.0× as long as wide. Frons with weak aciculations and punctures, and a weak median keel on lower half. Antennal funiculus with four or five segments, the last being short and wide. Antennal club with three straight or weakly procurved sutures. Pronotal colour dark brown. Pronotal profile broadly rounded, widest in line with summit. Pronotal margin armed with 6–8 wide, rather blunt serrations, larger at median. Pronotal declivity with 60–75 wide and blunt asperities (holotype has 71). Pronotal disc approx. three tenths of the pronotal length, asperate texture, most pronounced at median. Pronotal vestiture with golden hair-like setae, with some longer, coarse setae along baso-lateral margin. Suture between pronotum and elytra weakly sinuate. Scutellum small, triangular. Elytra very broadly rounded, disc occupying less than one third of elytral length, and a gradually sloping declivity. Elytra colour dark brown on disc, becoming chestnut-brown on declivity. Striae impressed on disc, gradually becoming less apparent and less impressed on declivity, visible only as row of shallow punctures on lower area of declivity. Interstrial bristles short, flattened near apex with a rounded tip, of a similar length on disc and declivity, curved pointing posteriorly, arranged somewhat irregularly on disc and in a row on declivity. Interstrial ground vestiture completely hair-like at base, barely indistinguishable from strial setae, becoming entirely tridentate scale-like on declivity. Protibiae and protarsi with only straight, hair-like setae. Mesocoxae moderately separated, a little more than distance between metacoxae. Ventrites with mostly single hair like, and some bifurcated setae. Proventriculus not examined.

**Male.** Similar to female except: Length 1.20–1.45 mm. Frons with minute transverse aciculations in the median upper portion of the frons. Interstrial ground vestiture tridentate scale-like on disc and declivity, more elongate and intermixed with a few hair-like setae near base. Protibiae and protarsi with a few coarse curved setae. Last abdominal ventrite not emarginated. Proventriculus sutural teeth numerous, occupying about half of width of segment, sometimes in indistinct transverse rows of three or more, with an indistinct transition to the apical teeth. Apical teeth almost extending width of segment. Closing teeth extending beyond masticatory brush, branched and finely tapered at tips. Masticatory brush of a similar length to apical plate. Aedeagus short, without obvious end plate. Penis apodemes less than half of the length of penis body. Tegmen with short paired apodemes.

###### Etymology.

The name is an adjective derived from a combination of *gnet* the stem scientific name of the host plant (*Gnetum*), a linking vowel -*i*- and an adjectival suffix *vorus*, meaning eater.

###### Distribution.

China (Guangdong).

###### Recorded plant hosts.

Gnetaceae: *Gnetum luofuense*C.Y. Cheng.

###### Remarks.

*Gnetum* is an unusual leafy gymnosperm distributed in Asia through to New Guinea, which grows as vines or small shrubs. *Cryphalus gnetivorus* is unusual among the *Cryphalus* in the area of study in the shape, with a robust pronotum and smaller elytra which is slightly tapered and mostly a gentle declivity, somewhat similar to *Eidophelus darwini* Eichhoff, 1878, or various Xyloctonini Eichhoff, 1878.

Using the key of Tsai and Li 1963, this species would key to “subgenus Cryphalus” and fail at couplet 2/7 if *Gnetum* is considered a conifer, and couplet 15/16 if *Gnetum* is considered a broadleaf. The colour and proportions are similar to *Cryphalus eriobotryae* Johnson, 2019, but can easily be distinguished by the antennal sutures (*C. gnetivorus*: evenly spaced and procurved; *C. eriobotryae*: unevenly distributed, the third much more procurved than first two), and using the diagnoses above.

##### Cryphalus itinerans

Taxon classificationAnimaliaColeopteraCurculionidae

Johnson sp. nov.

33A0AA54-AAEE-5F59-8F5E-ABC007153C6F

http://zoobank.org/A52892B8-430C-4113-9D1C-FCF118A06149

**Figure 9.**
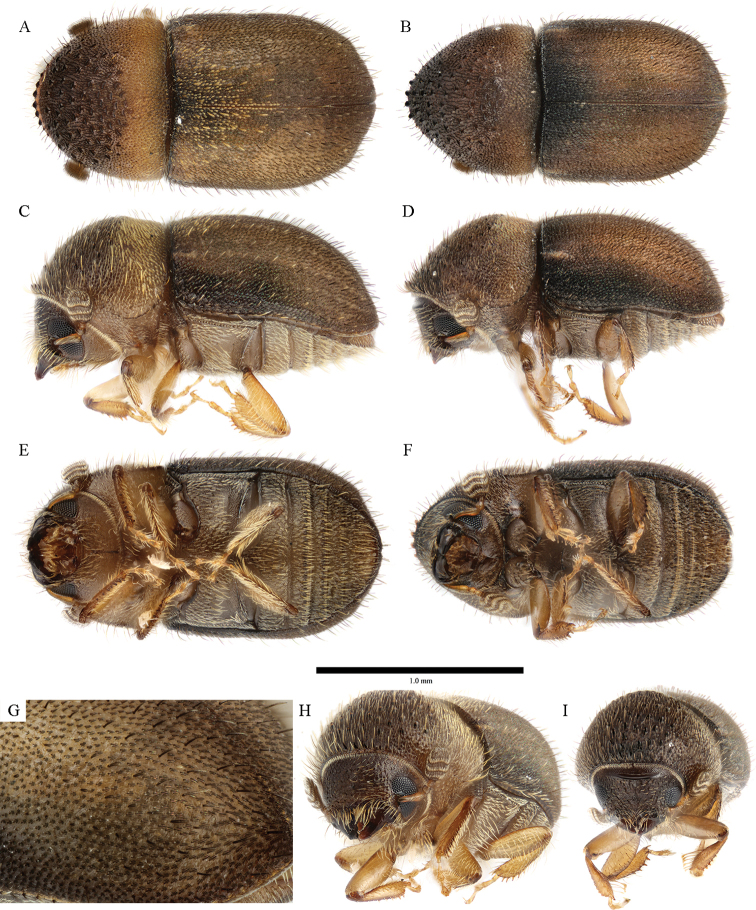
*Cryphalus itinerans***A, C, E, G, H** holotype, female UFFE:33170 **B, D, F, I** paratype, male, UFFE:31746.

Figures 2E , 3E , 9A–I

###### Type material examined.

China • 1 ♀ ***Holotype***; Hong Kong, Kadoorie Farm; 22.4294°N, 114.1146°E; Jun. 2017; James Skelton, P Carlson, You Li, and Jiri Hulcr leg.; IOZ(E)225673; ex vial 20306.; UFFE:33170; (IOZ) • 1 ♂ ***Paratype***; same collection data; ; dissected; DNA: 28S:MT431547, COI:MT431650; UFFE:33178; (UFFE) • 1 ♂ ***Paratype***; Hong Kong, Kadoorie Farm; 22.4294°N, 114.1146°E; Jun. 2017; James Skelton, P Carlson, You Li, and Jiri Hulcr leg.; UFFE:33174; (UFFE). .• 8 ♀♀, 8 ♂♂ ***Paratypes***; same collection data ; UFFE:33179; (NHMUK, 1♀, 1♂; FSCA, 1♀, 1♂; MZB, 1♀, 1♂; NIAES, 1♀, 1♂;NMNS, 1♀, 1♂; IOZ, 1♂ IOZ(E)225674; RIFID, 1♀, 1♂; USNM, 1♀, 1♂; ZIN, 1♀).

###### Other material examined.

China • 7; Fujian, Quanzhou, Yongchun, Diyiyan; 25.3177°N, 118.2809°E; 22 Nov. 2015; You Li leg.; beetle walking on the bark of *Broussonetia papyrifera*; DNA: 28S:MG051123, COI:MG051178; UFFE:22092; (UFFE) • 12; Guangdong, Shenzhen, Yantian; 22.5889°N, 114.2842°E; 06 Apr. 2017; Wei Lin leg.; UFFE:31618; (UFFE) • 2; Guangdong, Zhuhai, Jialinshan; 22.185°N, 113.4784°E; 05 Sep. 2018; Wei Lin, and You Li leg.:34039; (UFFE) • 2; Guangxi, Shangsi, Shiwandashan; 21.9278°N, 107.9282°E; 27 Mar. 2018; You Li, and Shengchang Lai leg.; near pine plantation; DAE; UFFE:34230; (UFFE) • 1 ♂; Hainan, Danzhou, near Botanic Garden; 19.5174°N, 109.4994°E; 22 Oct. 2016; You Li leg.; mix spp; DAE; UFFE:34187; (UFFE) • 1 ♂; Hainan, Qiongzhong, Wanling; 26 Oct. 2016; You Li leg.; Light trap; DNA: 28S:MG051124, COI:MG051179; UFFE:25935; (UFFE).

Taiwan • 7; Nantou County, Sun Moon Lake; 22 Apr. 2013; Ching-Shan Lin leg.; DNA: 28S:MG051126, COI:MG051180; UFFE:10482; (UFFE) • 1; Taichung City, Wufeng District, Jingtonlin; 24.0457°N, 120.788°E; 10 Jan. 2019; Ching-Shan Lin leg.; ex. *Ficuserecta Thunb. var. beecheyana*; UFFE:32769; (UFFE).

United States • 1 ♂; Florida, Escambia County, Pensacola, Ellyson industrial park; 30.5198°N, -87.2109°E; 20 Jul. 2012; J. Welch leg.; UFFE:15115; (FSCA) • 1 ♂; same collection data; DNA: 28S:MG051125; UFFE:15116; (UFFE) • 1 ♀; same collection data; UFFE:15117; (FSCA) • 1 ♀; Florida, Escambia County, Pensacola, Navy Point park; 21 May 2013; J. Brooks leg.; UFFE:15114; (FSCA).

###### Type locality.

China, Hong Kong, Kadoorie Centre (22.4294°N, 114.1146°E).

###### Diagnosis.

*Cryphalus itinerans* can be distinguished from most of the East Asian *Cryphalus* by the combination of the size (1.3–1.8 mm), by the pronotal summit, which is about a third of the length, viewed dorsally, and by abundant scales over all of the pronotal disc.

This species is very similar to *Cryphalus artocarpus* but differs by the size (*C. itinerans*: 1.3–1.8 mm, versus *C. artocarpus*: 1.05–1.30 mm), by the setae on the male protibiae (*C. itinerans*: scythe-shaped, versus *C. artocarpus*: hair-like, barely larger than on female), and by the proventriculus apical teeth (*C. itinerans*: more than two thirds segment width, versus *C. artocarpus*: less than two thirds segment width).

This species is also very similar to *C. dilutus* and can be diagnosed by the setae on the male protibiae (*C. itinerans*: scythe-shaped, versus *C. dilutus*: spatula-shaped); by the mesofemora of the males (*C. itinerans*: unarmed, versus *C. dilutus*: armed with a large spine).

**Female.** Length 1.40–1.80 mm (holotype 1.70 mm). Proportions 2.0× as long as wide. Frons with minute aciculations, barely visible. Antennal club with three procurved sutures marked by coarse and long setae. Antennal funiculus with four or five funicular segments (holotype has four). Pronotal colour dark brown on slope, light brown on disc. Pronotal profile broadly rounded, slightly wider in line with summit. Pronotal margin armed with five to eight serrations (holotype has six), separated by approximately their width, and sometimes flanked by one or two pairs of smaller serrations. Pronotal declivity with more than 50 asperities (holotype has 65). Pronotal disc approximately one third the length of the pronotum, gently sloped, weakly tuberculate surface texture (obscured by scale-like setae). Pronotal vestiture hair-like on anterior and lateral sloped, and a mixture of scale-like and hair-like on disc and postero-lateral regions, with the scale-like setae 2–3× as long as wide with a tridentate tip. Suture between pronotum and elytra weakly sinuate. Scutellum very small, barely visible. Elytra 1.5× as long as pronotum, translucent yellow-brown, broadly rounded with no clear transition to the declivity. Striae barely visible as rows of punctures and hair-like setae. Interstrial bristles erect, weakly flattened with rounded tips, some uniform in length and some wider near tip. Interstrial ground vestiture tridentate, approximately 1–2× as long as wide, translucent brown with a weak iridescence, sometimes light brown near the base of the elytra. Apex of elytra barely obtuse. Gular surface with evenly spaced hair-like setae. Protibiae and protarsi with only straight, hair-like setae. Protibiae and protarsi with only simple hair-like setae. Mesocoxae moderately separated, more than distance between metacoxae. Proventriculus not examined.

**Male.** Similar to female except: Length 1.30–1.70 mm. Frons weakly aciculate with a glossy carina above the level of the eyes. Gular surface shining around suture, surrounded by sparse setae. Pronotal profile widest in line with summit. Anterior to the summit, the profile is triangular. Pronotal margin with four to six marginal asperities, spaced approximately by twice their width. Pronotal declivity with almost straight slope, with more than 45 asperities, asperities smaller than the females. Pronotal disc with strongly tuberculate surface obscured by scale-like setae. Protibiae and protarsi with long scythe-shaped setae. Last abdominal ventrite clearly emarginated. Proventriculus sutural teeth rounded, in two overlapping rows. Apical teeth extending the width of a segment. Closing teeth very long, extending beyond masticatory brush, tapered, few branches. Masticatory brush short, less than length or apical plate. Aedeagus long, weakly sclerotised. Penis apodemes approximately two thirds as long as penis body. Tegmen with two ventral apodemes, which are longer than distance between them.

###### Etymology.

The name is derived from the Latin *itinerāns* meaning traveller, referring to the apparent ability to establish in new areas. It is invariable.

###### Distribution.

China (Hainan, Fujian, Guangdong, Yunnan, Hong Kong); Taiwan; United States (Florida).

###### Recorded plant hosts.

Moraceae: *Ficus carica* L., F. erecta Thunb. var. beecheyana, *Broussonetia papyrifera* (L.) Vent.

###### Suggested vernacular name.

Chinese: 华南梢小蠹 [South China twig beetle].

###### Remarks.

This species is weakly attracted to ethanol-quercivorol traps. It was observed making cave-like galleries in material 2–5 cm diameter.

This species was referred to as *Hypocryphalus* “sp.1422” in Johnson et al. (2017) where some of the distribution records and diagnostic characters were described. This species is abundant and widespread across Southern China and neighbouring regions. It is surprising that this species is not already described; it is perhaps because much of the work on *Cryphalus* in China has focused on species in the North or on coniferous hosts, as well as building upon work by researchers in the Russian Far East, not in tropical or sub-tropical regions. Several species from the Philippines such as *Cryphalus obesus* Hopkins, 1915 are similar but have a much shorter pronotal disc. *Cryphalus discretus* Eichhoff, 1878, from India and Myanmar, likely present in China, is also very similar, and differs by having a short elytral disc.

Using the key of Tsai and Li (1963), this species would be reach and match 23, *Cryphalus mandschuricus*, though the proportions and overall appearance differ greatly.

##### Cryphalus kyotoensis

Taxon classificationAnimaliaColeopteraCurculionidae

Nobuchi, 1966

0497FC22-D56D-5DFE-A602-12FFBA5306D1

**Figure 10.**
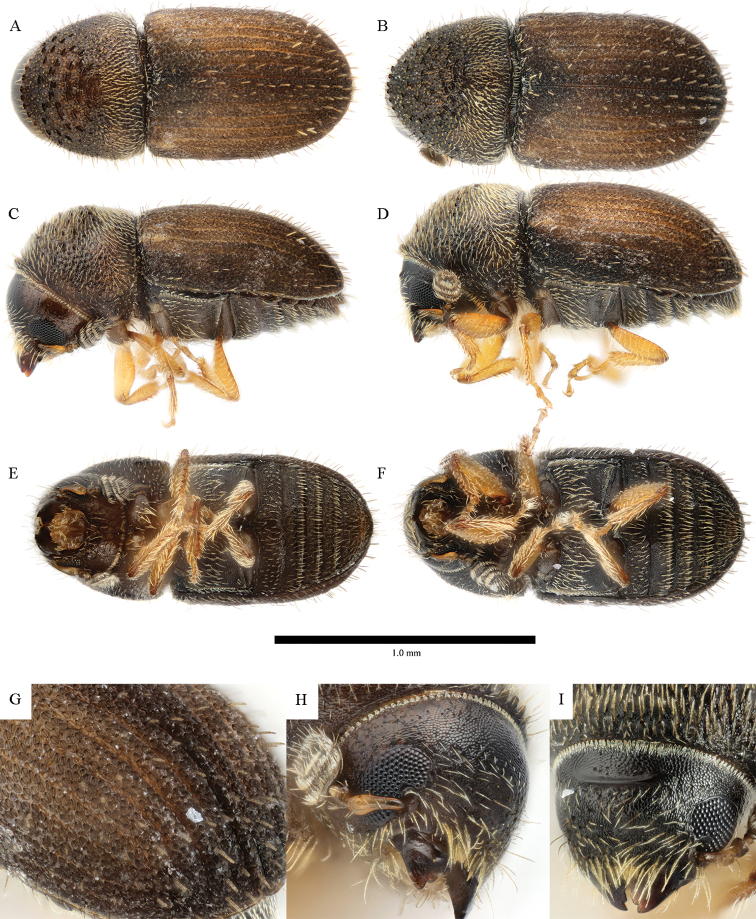
*Cryphalus kyotoensis***A, C, E, G, H** female UFFE:33170 **B, D, F, I** male, UFFE:31745.

Figures 2F , 3F , 10A–I

Cryphalus kyotoensis Nobuchi, 1966: 53 (Japan).

###### Type material examined.

Japan • 1 ♀ ***Holotype***; Kyoto, Kyoto City, Mizoro-ga-ike; 30 Dec. 1957; A. Nobuchi leg.; ex. *Alnus firma*; UFFE:34947 (NIAES).

###### Other material examined.

China • 1 ♀; Fujian, Longyan, Songmao Ridge, Liancheng; 25.57°N, 116.59°E; 03 Sep. 2019; Ling Zhang leg.; ex. *unknown*; 20190903001; AJJ also saw 1m and 1f from same collection, returned; UFFE:34068; (UFFE) • 1; Guangdong, Shenzhen, Yantian; 22.5889°N, 114.2842°E; 08 Apr. 2017; Wei Lin leg.; EtOH trap; UFFE:33203; (UFFE) • 1 ♀, 1 ♂; Jiangxi, Xunwu, Xiangshan; 24.9129°N, 115.8538°E; 10 Oct. 2018; You Li leg.; ex. *Rhus chinensis*; (IOZ, 1 ♀ IOZ(E)2057938, 1 ♂ IOZ(E)2057939); UFFE:31746; • 1 ♀; same collection data; DNA: 28S:MT431544, COI:MT431648; UFFE:33232 • 1 ♂ ; same collection data; UFFE:31745; (UFFE) • 9 ♀♀, 9 ♂♂ ; same collection data; (NHMUK, 1♀, 1♂; FSCA, 1♀, 1♂; MZB, 1♀, 1♂; NIAES, 1♀, 1♂; NMNS, 1♀, 1♂; RIFID, 1♀, 1♂; UFFE, 1♀, 1♂; USNM, 1♀, 1♂; ZIN, 1♀, 1♂); UFFE:31741.

South Korea • 1 ♀; Chungcheongbuk-do, Cheongju-si, Oksan-myeon, Guksa-ri; 02 Jul. 2008; Sangwook Park leg.; UFFE:28140; (UFFE) • 1 ♂; same collection data; DNA: 28S:MT431543, COI:MT431647; UFFE:28139; (UFFE) • 5; same collection data; UFFE:28138; (UFFE) • 65 ♀♀, 77 ♂♂; Chungcheongnam-do, Gongju-si, Banpo-myeon, Maam-ri; 36.4252°N, 127.1979°E; 02 Aug. 2009; Sangwook Park leg.; UFFE:34708; (RIFID) • 1 ♀, 1 ♂; Gangwon-do, Chuncheon-si, Dong-myeon, Gamjeong-ri; 37.8952°N, 127.8218°E; 04 May 2017; Sangwook Park leg.; UFFE:34705; (RIFID) • 1 ♀, 2 ♂♂; Gangwon-do, Goseong-gun, Toseong-myeon, Wonam-ri; 38.2136°N, 128.4707°E; 15 Apr. 2016; Sangwook Park leg.; UFFE:34698; (RIFID) • 1 ♂; Gangwon-do, Inje-gun, Inje-eub, Nambuk-ri; 38.0753°N, 128.1336°E; 23 Apr. 2013; Sangwook Park leg.; UFFE:34701; (RIFID) • 2 ♀♀, 7 ♂♂; Gangwon-do, Inje-gun, Buk-myeon, Hangye-ri; 38.1357°N, 128.2763°E; 17 Apr. 2018; Sangwook Park leg.; UFFE:34702; (RIFID) • 2 ♀♀, 4 ♂♂; Gangwon-do, Inje-gun, Buk-myeon, Hangye-ri; 38.1357°N, 128.2763°E; 08 May 2018; Sangwook Park leg.; UFFE:34707; (RIFID) • 1 ♂; Gangwon-do, Samcheok-si, Mapyeong-dong; 37.4244°N, 129.1345°E; 12 Sep. 2013; Sangwook Park leg.; UFFE:34696; (RIFID) • 1 ♂; Gangwon-do, Samcheok-si, Mapyeong-dong; 37.4268°N, 129.1351°E; 16 Jun. 2016; Sangwook Park leg.; UFFE:34704; (RIFID) • 3 ♀♀; Gangwon-do, Yangyang-gun, Seo-myeon, Yeongdeok-ri; 37.9914°N, 128.5304°E; 15 Apr. 2016; Sangwook Park leg.; UFFE:34700; (RIFID) • 1 ♂; Gangwon-do, Yangyang-gun, Seo-myeon, Seorim-ri; 37.9634°N, 128.5178°E; 17 Apr. 2018; Sangwook Park leg.; UFFE:34697; (RIFID) • 1 ♂; Gangwon-do, Yangyang-gun, Seo-myeon, Osaek-ri; 38.0746°N, 128.4818°E; 08 May 2018; Sangwook Park leg.; UFFE:34699; (RIFID) • 6 ♂♂; Gyeonggi-do, Gapyeong-gun, Seolak-myeon, Seonchon-ri; 37.6769°N, 127.4716°E; 15 Apr. 2016; Sangwook Park leg.; UFFE:34706; (RIFID) • 1; Gyeongnam-do, Sangcheong-gun, Sicheon-myeon, Jungsan-ri; 35.1802°N, 127.4511°E; 01 Aug. 2008; Sangwook Park leg.; UFFE:28143; (UFFE) • 8 ♀♀, 6 ♂♂; Gyeongsangbuk-do, Sangju-si, Sabeol-myeon, Deokga-ri; 36.5091°N, 128.2241°E; 13 Apr. 2018; Sangwook Park leg.; UFFE:34695; (RIFID) • 1 ♀; Gyeongsangbuk-do, Sangju-si, Sabeol-myeon, Deokga-ri; 36.5091°N, 128.2241°E; 25 Apr. 2018; Sangwook Park leg.; UFFE:34703; (RIFID) • 1; Gyeongsangnam-do, Sangcheong-gun, Sicheon-myeon, Jungsan-ri; 35.1802°N, 127.4511°E; 01 Aug. 2008; Sangwook Park leg.; UFFE:28142; (UFFE).

###### Diagnosis.

This species can be identified by the combination of the size (1.10–1.30 mm), the proportions (2.15× as long as wide) and the scale-like interstrial ground vestiture which is fused to the elytra.

**Female.** Length 1.20–1.30 mm (holotype 1.15 mm). Proportions 2.15× as long as wide. Frons simple, convex, with a small fovea in the centre. Antennal club with three recurved sutures marked by coarse setae. Antennae with three funicular segments. Pronotal colour dark brown. Pronotal profile widest at base, broadly rounded. Pronotal margin armed with eight serrations. Pronotal declivity with approx. 40–50 asperities, some of which are joined near the summit. Pronotal disc approximately one third of the pronotal length, sloping weakly from the summit. Pronotal vestiture coarse hair-like, light golden brown. Suture between pronotum and elytra weakly sinuate, marked with a carina at the base of the pronotum. Scutellum V-shaped, with hair-like setae. Elytra 1.6× as long as pronotum, translucent yellow-brown, broadly rounded with no clear transition to the declivity. Striae clearly visible as rows devoid of ground vestiture, and weakly visible punctures. Interstrial bristles erect, hair-like with blunt tips. Interstrial ground vestiture scale-like, recumbent, appearing fused to the elytra, sitting convex with a median keel; less than 1.5× as long as wide. Protibiae and protarsi with only straight, hair-like setae. Mesocoxae separated, barely more than metacoxae. Proventriculus not examined.

**Male.** Similar to female except: Length 1.10–1.30 mm. Frons with a slightly deeper fovea, and a distinct transverse carina above the level of the eyes. Last abdominal ventrite not emarginated, similar to female. Proventriculus sutural teeth irregularly sized and shaped, though mostly rounded/conical, in two or more confused rows. Apical teeth extending only about half of segment width. Closing teeth long, barely branched near tip. Masticatory brush shorter than apical plate. Aedeagus long, bulbous at the base. Penis apodemes longer than penis body. Tegmen without paired apodemes.

###### Distribution.

China (Fujian, Guangdong, Jiangxi); Japan; South Korea.

###### Ecology.

Collected from branches 4–10 cm diameter.

###### Recorded plant hosts.

Anacardiaceae: *Rhus chinensis* Mill.; Betulaceae: *Alnus firma* Siebold & Zucc.

###### Remarks.

This species is phylogenetically similar to *C. longus* Eggers, 1926 from Primorskiy Kray and Japan (and likely present in northern China), which shares the unusual recumbent setae, but differs in the proportions.

The aedeagus is somewhat unusual among *Cryphalus* for having no paired apodemes on the tegmen, which is usually present and unique to *Cryphalus*.

##### Cryphalus lipingensis

Taxon classificationAnimaliaColeopteraCurculionidae

Tsai & Li, 1959

E8F20202-6634-582B-A106-2227F5D153B2

**Figure 11.**
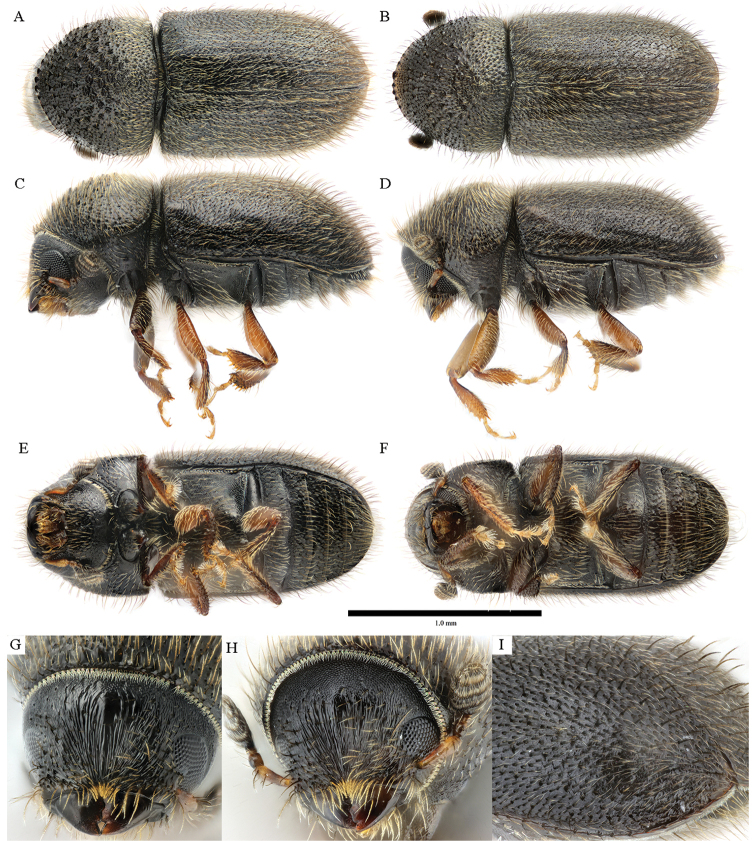
*Cryphalus lipingensis***A, C, E, G** female, UFFE:28042 **B, D, F, H, I** male, UFFE:28041.

Figs 2G , 3G , 11A–I

Cryphalus lipingensis Tsai & Li, 1959: 90 (China).
Cryphalus kesiyae Browne, 1975: 288 (Thailand), syn. nov.

###### Type material examined.

China • 1 ♂ ***Lectotype*** [here designated]; 陕西黎坪 [Shaanxi, Liping town]; 15 Jun. 1958; 宋士美[Shimei Song leg].; 华山松 [*Pinus armandii*]; IOZ(E) 213228; UFFE:34756; (IOZ) • 33 ***Paralectotypes*** ; same collection information and labels; IOZ(E) 213218 to 213221; 213223 to 213250, 213436, and 213437; UFFE:34755; (IOZ).

Thailand • 1 ♂ ***Holotype****Cryphalus kesiyae* Browne, 1975; Chang Mai, Doi Pui; 15 Feb. 1971; Roger A. Beaver leg.; ex. *Pinus kesiya*; NHMUK 010805965; UFFE:26208; (NHMUK) • 1 ♀ ***Paratype***; Chang Mai, Doi Pui; 15 Feb. 1971; Roger A. Beaver leg.; ex. *Pinus kesiya*; UFFE:10407; (NHMUK).

###### Other material examined.

China • 1 ♂; Guizhou, Weining, Maanshan; 26.822°N, 104.6282°E; 20 Oct. 2015; You Li leg.; ex. *Pinus armandii*; dissected; UFFE:28048; (UFFE) • 1 ♂; same collection data; UFFE:28041; (UFFE) • 1 ♀; same collection data; UFFE:28042; (UFFE) • 11 ♀♀, 12 ♂♂; Guizhou, Weining, Meihua village; 26.7251°N, 104.6027°E; 16 Oct. 2015; You Li leg.; ex. *Pinus armandii*; reared from multiple branches; (NHMUK, 1♀, 1♂; FSCA, 1♀, 1♂; MZB, 1♀, 1♂; NIAES, 1♀, 1♂; NMNS, 1♀, 1♂; IOZ, 1 ♀ IOZ(E) 2057940, 1 ♂ IOZ(E) 2057941; RIFID, 1♀, 1♂; UFFE, 2♀♀, 3♂♂; USNM, 1♀, 1♂; ZIN, 1♀, 1♂); UFFE:32956 ; same; same• 1 ♂; Yunnan, Kunming; 24.8801°N, 102.8329°E; 30 May 2013; Bateman leg.; UFFE:10939; (UFFE).

Thailand • 1; Chang Mai, Nanthaburi District, Omkoi Wildlife Sanctuary; 28 Jun. 2013; Bateman leg.; DNA: 28S:MG051085, COI:MG051133; UFFE:13592; (UFFE) • 1; Chiang Mai, Nanthaburi District, Omkoi Wildlife Sanctuary; 28 Jun. 2013; Bateman leg.; from vial 6101; voucher; UFFE:28529; (UFFE).

###### Diagnosis.

This species can be easily distinguished from all other Chinese species by the combination of the strong aciculations on frons, by the male frons without transverse ridge or sulcus, by the long hair-like ground vestiture (only slightly widened at the base) and by the proventriculus with single row of sutural teeth.

###### Distribution.

China (Guizhou, Sichuan, Shaanxi, Yunnan); Thailand.

**Female.** Length 1.70 mm (types 1.5–1.9 mm). Proportions 2.25× as long as wide. Frons distinctly aciculate, converging to the epistoma. Antennal club with three weakly recurved sutures marked by coarse and long setae, the distance between the suture 3 and the apex similar to the distance between sutures 2 and 3. Antennal funiculus with four segments, length shorter than the scape. Gular surface with evenly spaced hair-like setae. Pronotal colour dark brown, similar colour to elytra. Pronotal profile broadly rounded, slightly triangular, widest in line with summit, approx. 0.8× as long as wide. Pronotal margin armed with six serrations, often irregular and asymmetrical in size. Pronotal declivity with more than 70 asperities. Pronotal disc approximately one quarter the length of the pronotum, sloped, weakly tuberculate surface texture, larger tubercles near summit. Pronotal vestiture entirely hair-like setae. Suture between pronotum and elytra weakly sinuate. Scutellum very small, barely visible. Elytra 1.95× as long as pronotum, orange brown to brown, broadly rounded with no clear elytral disc or a transition to the declivity. Striae barely visible as rows of punctures and slightly impressed. Interstrial bristles erect, hair-like, with pointed tips, slightly longer and more dense on the declivity Interstrial ground vestiture hair like with pointed tips, denser on declivity. Protibiae and protarsi with only straight, hair-like setae. Mesocoxae moderately separated, a little more than distance between metacoxae. Ventrites each with tooth on the postero-lateral corners. Proventriculus not examined.

**Male.** Similar to female except: Length 1.50–1.70 mm. Proportions 2.30× as long as wide. Frons convex, without transverse ridge or sulcus. Gular surface simple with few hair-like setae. Pronotal declivity more flat than female. Protibiae and protarsi with only hair-like setae, almost the same as female. Last abdominal ventrite weakly emarginated. Proventriculus sutural teeth small, in a single row on either side of the suture. Apical teeth extend the width of the entire segment, of typical proportions in the median. Closing teeth mostly shorter than length of masticatory brush, barely branched, and rounded tips. Masticatory brush about half of total length. Aedeagus short. Penis apodemes about as long as penis body, fused at tip. Tegmen with paired apodemes much shorter than distance between. End plate barely visible as two sclerotised plates.

###### Recorded plant hosts.

Pinaceae: *Pinus kesiyae*, *P. armandi*, *P. yunnanensis*.

###### Suggested vernacular name.

Chinese: 华山松梢小蠹 [Huashan pine twig bark beetle] (Tsai and Li 1959).

###### Remarks.

We examined the holotype and paratypes of *Cryphalus kesiyae* Browne, 1975, and photographs of types of *C. lipingensis*, plus specimens from Thailand and China. There is little variation among the specimens including the type material, and more recent exemplars from near the type localities are genetically similar. Despite being from a similar region, Browne (1975) did not mention the similar species, but the descriptions were in Chinese and all of the large type series was held in China.

It appears that a holotype for *Cryphalus lipingensis* was never designated. The original description included two localities, “Liping”, Shaangxi Province, from which it was named, and also Nanjiang, Sichuan Province. All of the specimens at IOZ were labelled as paratypes with a printed yellow label “PARATYPE”, and all are from “Liping”, presumed as the intended type locality. A similar issue exists for the other species described by Tsai and Li in 1959, where no holotype is located or even mentioned. The specimens labelled as paratypes are therefore assumed to be syntypes, and to promote stability, a lectotype for *Cryphalus lipingensis* is hereby designated as specimen with the label “IOZ-(E) 213228”.

##### Cryphalus mangiferae

Taxon classificationAnimaliaColeopteraCurculionidae

Stebbing, 1914

C033FC35-C25B-55EF-8004-DE6F0194772A

**Figure 12.**
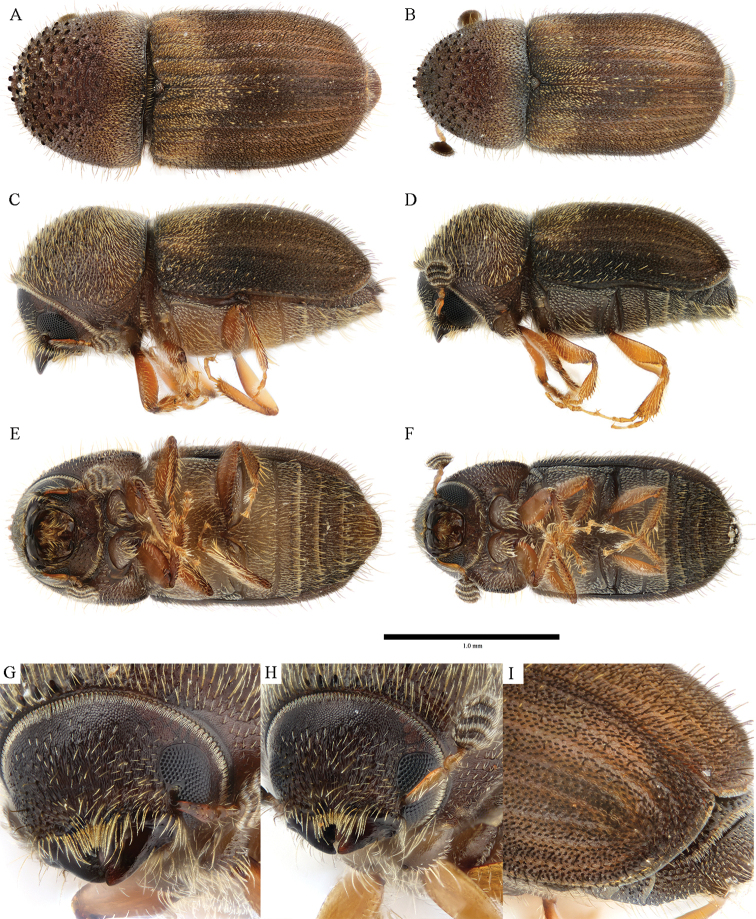
*Cryphalus mangiferae***A, C, E, G** female, UFFE:33207 **B, D, F, H, I** male, UFFE:33208.

Figures 2H , 3H , 12A–I

Cryphalus inops Eichhoff, 1872: 131 (Guadeloupe).
Hypothenemus griseus Blackburn, 1885: 194 (Hawaii).
Cryphalus mangiferae Stebbing, 1914: 542 (India).
Hypocryphalus mangiferae Eggers, 1928: 85 (Brazi).
Cryphalus subcylindricus Schedl, 1942: 16 (Indonesia).
Cryphalus mimicus Schedl, 1942: 17 (Indonesia).
Hypocryphalus opacus Schedl, 1942; 20 (Indonesia).
Taenioglyptes artestriatus Browne, 1970: 553 (Uganda), syn. nov.

###### Type material examined.

India • 1 ♀ ***Lectotype****Cryphalus mangiferae* Stebbing, 1914; 1902; labelled “India// E.P.Stebing//902–309. //// mango twigs // *Cryphalus mangiferae* Stb. // C. Beeson det. //// Lectotype *Cryphalus mangiferae* // S.L.W. 1976 Stebb. ////NHMUK 010805929”; UFFE:26286; (NHMUK) • 1 ♂? ***Paralectotype***; 1902; UFFE:10426; (NHMUK).

Uganda • 1 ♀ ***Holotype****Taenioglyptes artestriatus* Browne, 1970; Central Region, Wakiso, Zika; 0.12°N, 32.52°E; 21 May 1961; K. W. Brown leg.; “***Holotype***////Uganda//Zika//in light trap//K. W. Brown //29/5/61 //B.1715(g) ////1666 ////Taenioglyptes artestriatus// Browne ♀ //***Holotype***////Brit. Mus. 1973–70////NHMUK 010806013”; UFFE:26192; (NHMUK).

###### Other material examined.

China • 1 ♀; Fujian, Quanzhou, Nan’an, Pushan village; 25.1189°N, 118.4276°E; May 2017; You Li leg.; ex. *Mangifera indica*; UFFE:33207; (UFFE) • 1 ♂; same collection data; UFFE:33208; (UFFE) • 9 ♀♀, 8 ♂♂; same collection data; UFFE:33206; (NHMUK, 1♀, 1♂; FSCA, 1♀; MZB, 1♀, 1♂; NIAES, 1♀, 1♂; NMNS, 1♀, 1♂; IOZ 1♀ IOZ(E)2057932, 1♂ IOZ(E)2057933; RIFID, 1♀, 1♂; USNM, 1♀, 1♂; ZIN, 1♀, 1♂) • 1 ♂; same collection data; dissected; UFFE:34966 (UFFE); • 1 ♀; Hunan, Changsha, Yuelushan; Aug. 2019; You Li leg.; ex. *Choerospondias axillaris*; DNA: 28S:MT431546; UFFE:33551 • 57; Hunan, Changsha, Yuelushan; 28.1897°N, 112.9329°E; Aug. 2019; You Li leg.; ex. *Choerospondias axillaris*; UFFE:33529; (UFFE) • 1; Yunnan, Mengla XTBG; C. Bateman leg.; DNA: 28S:MG051112, COI:MG051163; UFFE:14119; (UFFE) • 1; Yunnan, Menglun XTBG; 20 Jul. 2017; Craig Bateman leg.; DNA: COI:MG051162; UFFE:25419; (UFFE).

Guadeloupe • 1 ♂; Basse Terre, Point a Lezard; 20 May 2012; R. Turnbow leg.; UFFE:12501; (FSCA).

Taiwan • 1; Kaohsiung, Liouguei; Tom Harrington, and Caroline Wuerst leg.; Lindgren trap with ethanol; DNA: 28S:MG051118, COI:MG051172; UFFE:15127; (UFFE).

United States • 1; Florida, Homestead; 29 Jun. 2015; Thomas H. Atkinson leg.; ex. *Mangifera indica*; from vial 9444; DNA: 28S:MG051119, COI:MG051174; UFFE:25414; (UFFE) • 18; Florida, Miami Dade, Homestead, TREC; 23 Jul. 2018; Andrew J. Johnson leg.; ex. *Mangifera indica*; UFFE:29770; (UFFE). • 1; Florida, Dade County, Homestead; 2012-Apr-05; L. Bradshaw leg.; ex. *Codiaeum variegatum*.

Vietnam • 1 ♀; TamDao; Michelle Jusino, and James Skelton leg.; quercivorol and ethanol trap; DNA: 28S:MG051120, COI:MG051175; UFFE:25936; (UFFE).

###### Diagnosis.

This species is distinguished from other similar *Cryphalus* by the frons with a finely aciculate texture, the pronotal disc which is long, and has coarse hair-like setae, the elytral striae which are barely impressed but apparent by the rows without ground vestiture, and by the shape of ground vestiture which have tapered tips.

See notes for diagnosing the very similar *Cryphalus paramangiferae*, described below, under the diagnosis for that species.

**Female.** Length 1.6–2.2 mm. Proportions 2.2× as long as wide. Frons with weak converging aciculations. Antennal funiculus with four or five funicular segments. Antennal club with three evenly procurved sutures mostly marked by coarse setae. Pronotal profile widest in line with summit. Pronotal margin armed with four to six widely spaces serrations, the median pair larger. Pronotal declivity with more than 60 asperities. Pronotal disc approximately one third of the length, gently sloped. Pronotal vestiture entirely hair-like or dagger-like, very few setae bifurcating in baso-lateral area Suture between pronotum and elytra weakly sinuate. Scutellum shaped as a rounded triangle, almost semi-circular, with sparse, pale, hair-like setae. Elytra 1.6× as long as pronotum, usually a similar colour to pronotum, translucent yellow-brown, sometimes to dark brown, broadly rounded with no clear transition to the declivity. Striae weakly visible as rows of punctures and hair-like setae with almost no ground vestiture, barely impressed Interstrial bristles erect, curving posteriorly, of approximately even diameter. Interstrial ground vestiture near triangular, dagger-like, tapering to a fine point, longer on declivity, ground vestiture on basal third are usually a light brown/cold colour. Mesocoxae moderately separated, much more than metacoxae. Ventrites with mostly hair-like setae. Last abdominal ventrite with margin of rounded tubercles Proventriculus sutural teeth weakly sclerotised, rounded, irregular, one indistinct row each side of suture. Apical teeth in multiple rows, extending almost the width of a segment. Closing teeth long, barely branched. Masticatory brush short, less than length or apical plate.

**Male.** Similar to female except: Length 1.5–2.2 mm. Frons identical to females, with granulate texture on median of upper level. Pronotal profile slightly more triangular, widest nearer base. Pronotal vestiture hair like, with a few bifurcating setae on baso-lateral areas, slightly more than on female. Protibiae and protarsi with several larger, coarse curved setae on the proximal edge near the apex. Last abdominal ventrite weakly emarginated. Aedeagus long, penis body sclerotised, tapered to a point at apex, ejaculatory duct with small, evenly sized spinulae, end plates sclerotised. Penis apodemes 2.5× as long as penis body. Tegmen broad, with very small, almost obsolete paired apodemes.

###### Distribution.

China (Fujian, Guangdong, Hunan, Yunnan); Taiwan; Thailand; Malaysia; Indonesia; India; Nepal; United States (Florida, Hawaii); Mexico; Puerto Rico; Cuba; Kenya, Uganda; American Samoa, Australia.

###### Suggested vernacular name.

Chinese: 芒果梢小蠹 [Mango twig bark beetle]; English: Mango bark beetle.

###### Recorded plant hosts.

Anacardiaceae: *Mangifera indica* L., *M. odorata* Griff. (Kalshoven 1958), *Choerospondias axillaris* (Roxb.) B.L.Burtt & A.W.Hill; Euphorbiaceae: *Codiaeum variegatum* (L.) A.Juss. [not confirmed reproductive host].

###### Remarks.

*Choerospondias axillaris* is a specialty crop in southern China, and it represents a new reproductive host record. A record from *Codiaeum variegatum* from Florida suggests that the beetle might have a broader host range than previously thought, though *Codiaeum* was not confirmed as a reproductive host. Kalshoven (1958) also lists *Zizyphus oenopila* (L.) Mill. (Rhamnaceae), *Theobroma cacao* L. (Malvaceae), *Lansium parasiticum* (Osbeck) Sahni & Bennet (Meliaceae) and *Canarium commune* L. (Burseraceae), but the associated vouchers were not examined for accuracy. Beaver (1990) also recorded this species from an unknown angiosperm not *Mangifera*. No host outside of Anacardiaceae has been verified as a reproductive host.

The holotype of *Taenioglyptes artestriatus* Browne, 1970, while not from the regions of study, is recognised as a synonym. All diagnostic characters are visible. The antennae have four funicular segments, which previously distinguished the genera *Cryphalus* and *Hypocryphalus*, probably leading to this error. Several examples from China had similar antennae, and one specimen from Guadeloupe.

##### Cryphalus meridionalis

Taxon classificationAnimaliaColeopteraCurculionidae

(Nobuchi, 1975)

E27EEA20-B844-5A6A-86A0-8DBABCE31136

**Figure 13.**
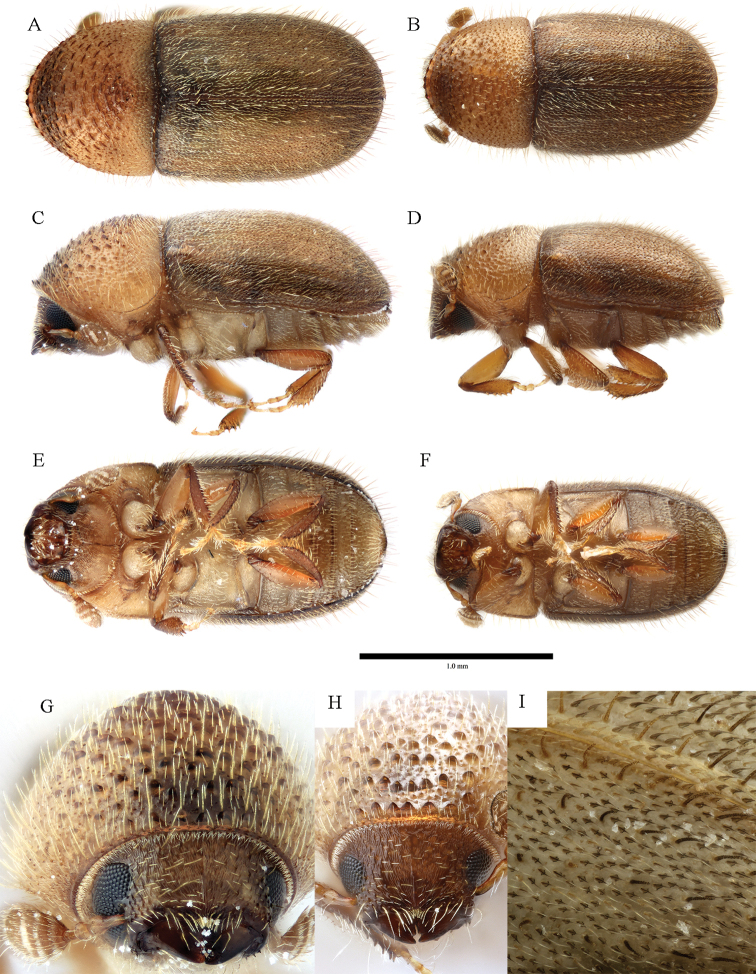
*Cryphalus meridionalis***A, C, E, G** female, UFFE:28062 **B, D, F, H** male, UFFE:34962 **I** female, UFFE:33236.

Figures 2I , 3I , 13A–I

Taenioglyptes meridionalis Nobuchi, 1975: 55 (Japan).

###### Type material examined.

Japan • 1 ***Holotype***; Okinawa, Kunigami District, Yona Experimental Forest; 26.763°N, 128.216°E; 08 Jul. 1965; K. Takahashi leg.; UFFE:28614; (NIAES).

###### Other material examined.

China • 1 ♂ ; Fujian, Quanzhou, Yongchun, Diyiyan; 25.3176°N, 118.279°E; 02 May 2017; You Li leg.; ex. *Schefflera heptaphylla*; DNA: 28S:MT122095; UFFE:28061; (UFFE) • 1 ♀ ; same collection data; UFFE:28062; (UFFE) • 1 ♂ ; same collection data; UFFE: 34962; (UFFE) • 1 ♀, 1 ♂; same collection data; (IOZ, 1 ♀ IOZ(E)2057942, 1 ♂ IOZ(E)2057943) • 1; Fujian, Zhangzhou, Yunxiao, Jiangjunshan Mt.; 23.952°N, 117.312°E; 25 Jul. 2019; Ling Zhang leg.; light trap with EtOH; 20190725–28001; UFFE:34069; (UFFE).

Japan • 1; Okinawa, Kunigami District, Yona Experimental Forest; 26.763°N, 128.216°E; Nov. 2010; Jiri Hulcr leg.; ex. Moraceae ?; teneral adult; DNA: 28S:MT431537; UFFE:07484; (UFFE)• 1 ♀; same collection data; UFFE:33236; (UFFE).

###### Diagnosis.

*Cryphalus meridionalis* can be distinguished from other East Asian *Cryphalus* by the barely aciculate frons with a fine sharp median keel, a short transverse sulcus of the male frons, by the pronotal slope with wide, barely protruding asperities, by the short pronotal disc, by the ground vestiture of which the setae are widened near the base and hair-like at the tips, and by the proventriculus without any sutural teeth.

**Female.** Length 1.70–2.10 mm (holotype 1.80 mm). Proportions 2.1× as long as wide. Frons aciculate, converging to the epistoma, with a weak median keel. Antennal club with three weakly procurved sutures marked by coarse and long setae, the distance between the suture 3 and the apex approximately twice the distance between sutures 2 and 3. Antennal funiculus with four segments, length shorter than the scape. Gular surface with evenly spaced hair-like setae. Pronotal colour orange-brown to brown, slightly lighter than elytra. Pronotal profile broadly rounded, slightly triangular, widest in line with summit, approx. 0.75× as long as wide. Pronotal margin armed with six to eight serrations, the median pair or a similar size to the others. Pronotal declivity with more than 70 asperities (holotype has approx. 77), each wide and barely protruding. Pronotal disc approximately one quarter the length of the pronotum, sloped, weakly tuberculate surface texture. Pronotal vestiture entirely hair-like setae. Suture between pronotum and elytra weakly sinuate. Scutellum very small, barely visible. Elytra 1.8 × as long as pronotum, orange brown to brown, broadly rounded with no clear elytral disc or a transition to the declivity. Striae not apparent. Interstrial bristles erect, hair-like, with rounded tips, slightly shorter on disc. Interstrial ground vestiture wide at base, with tapered, hair-like tips. Protibiae and protarsi with only straight, hair-like setae. Mesocoxae moderately separated, more than distance between metacoxae. Proventriculus not examined.

**Male.** Similar to female except: Length 1.65–2.00 mm Proportions 2.0× as long as wide Frons with converging aciculations and a fine median keel up to a short carina and shining sulcus above the level of the eyes. Gular surface simple with few hair-like setae. Pronotal profile rounded triangular, protruding slightly more than of the female. Pronotal declivity more flat than in female, with smaller asperities. Protibiae and protarsi with only hair-like setae, almost the same as female. Last abdominal ventrite emarginated. Proventriculus sutural teeth completely absent. Proventriculus apical teeth coarse, the median teeth are wider and rounded Proventriculus closing teeth mostly shorter than length of masticatory brush, barely branched, and rounded tips Proventriculus masticatory brush about half of total length Aedeagus short. Penis apodemes longer than penis body, fused at tip. Tegmen with paired apodemes shorter than distance between. End plate visible as two sclerotised plates.

###### Distribution.

China (Fujian), Indonesia (Java), Japan (Ryukyu Islands)

###### Remarks.

The original spelling of the name, as “*merdionalis*”, was used throughout the original description. This was amended to “*meridionalis*” in subsequent publications, the correct spelling to refer to the species distribution in the southern region of Japan. No explicit correction was made following correct procedures (ICZN art. 33.2.1), but in all subsequent publications, with exception to a website listing types, the amended spelling is used clearly referencing the original publication, and this is considered sufficient prevailing usage under 33.2.3.1 to consider the change a justified emendation.

###### Recorded plant hosts.

Araliaceae: *Schefflera* spp., “Moraceae”[?]

##### Cryphalus morivorus

Taxon classificationAnimaliaColeopteraCurculionidae

Johnson sp. nov.

C9A2A75E-AA02-52B1-83B7-BFAC4A9A1E25

http://zoobank.org/2CA70B2D-F6BA-441C-B6D0-5CF60A0553EB

**Figure 14.**
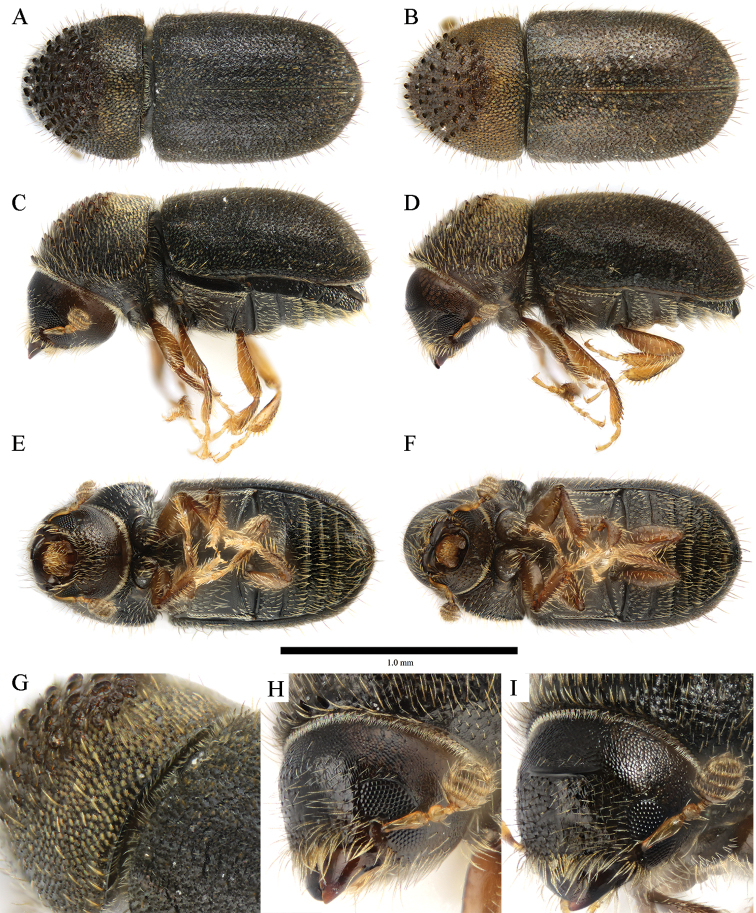
*Cryphalus morivorus***A, C, E, G, H** holotype, female, UFFE:31722 **B, D, F, I** paratype, male, UFFE:31723.

Figures 2J , 3J , 14A–I

###### Type material examined.

China • 1 ♀ ***Holotype***; Hebei, Chengde, Shuangqiao; 41.021°N, 117.943°E; 08 Jul. 2017; You Li leg.; ex. *Morus*; IOZ(E)225669; UFFE:31722; (IOZ) • 1 ♂ ***Paratype***; same collection data; UFFE:31723; (UFFE) • 14 ♀♀, 13 ♂♂ ***Paratypes***; same collection data; UFFE:34757; (NHMUK, 1♀, 1♂; FSCA, 1♀, 1♂; MZB, 1♀, 1♂; NIAES, 1♀, 1♂; NMNS, 1♀, 1♂; IOZ, 1♂ (IOZ(E)225670); RIFID, 1♀, 1♂; UFFE, 6♀♀, 3♂♂; USNM, 1♀, 1♂; ZIN, 1♀, 1♂) • 1 ♂ ***Paratype***; Hebei, Chengde, Shuangqiao; 41.021°N, 117.943°E; Aug. 2017; You Li leg.; ex. *Morus*; field notes: mulberry fresh cut twig with green bark; specimen dissected; DNA: 28S:MT431541, COI:MT431646; UFFE:27606; (UFFE).

###### Other material examined.

China • 1 ♀; Hebei, “Peking” [Beijing], “Pataling” [Badaling]; 11 Jul. 1972; Fusheng Huang leg.; ex. *Morus alba*; UFFE:12190; (USNM) • 1; Hebei, Chengde, Shuangqiao; 41.021°N, 117.943°E; Aug. 2017; You Li leg.; ex. *Morus*; dissected; DNA: 28S:MT122093; UFFE:27605; (UFFE) • 1 ♂; same collection data; UFFE:27604; (UFFE) • 1 ♀; 河北蓟县 [Hebei, Jixian]; 21 Jun. 1993; 于丽辰采集 [Lichen Yu leg.]; 桑树[*Morus*]; UFFE:33460; (IOZ) • 1 ♂; 河北蓟县 [Hebei, Jixian]; 21 Jun. 1993; 于丽辰采集 [Lichen Yu leg.]; 桑树 [*Morus*]; IOZ(E)701629; UFFE:33461; • 2; Shandong, Taian, Shandong Agricultural University campus; 36.168°N, 117.1542°E; 16 Oct. 2018; You Li leg.; ex. *Morus alba*; from twig, old cut; UFFE:31712; (UFFE) • 3; Shanghai, Shanghai Academy of Landscape Architecture Science and Planning; 31.1527°N, 121.4493°E; Dec. 2017; Lei Gao leg.; ex. *Morus*; UFFE:29907; (UFFE).

South Korea • 1 ♂; “경남 함양 마천 백무동” [Gyeongsangnam-do, Hamyang-gun, Macheon-myeon, Baekmudong valley]; 07 Nov. 1982; “추 호 렬” [Choo Ho Yul leg.]; “기주: 뽕나무” [ex. *Morus alba*]; UFFE:34726; (UFFE) • 1 ♂; same collection data; UFFE:34727; (UFFE) • 6 ♂♂; same collection data; UFFE:34782; (RIFID).

Taiwan • 1 ♂; Taichung, Taichung, Caohu; 24.079°N, 120.6903°E; 02 May 2018; Ching-Shan Lin leg.; ex. *Morus australis*; UFFE:33336; (UFFE) • 1 ♂; same collection data; DNA: 28S:MT431542; UFFE:33337; (UFFE).

###### Type locality.

CHINA, Hebei, Chengde, Shuangqiao, 41.0210°N, 117.9430°E.

###### Diagnosis.

This species can be distinguished from other *Cryphalus* in East Asia by the size (1.35–1.70 mm), by the proportions (2.25× as long as wide), by the male frons with a transverse carina, by the many scale-like setae on the pronotal disc extending to the lateral margins, and by the barely apparent elytral striae.

*Cryphalus morivorus* is very similar to *C. exiguus* and can be distinguished by the pronotal disc (*C. morivorus*: covered by scale-like setae, versus *C. exiguus* with entirely coarse hair-like setae), by the elytral striae (*C. morivorus*: barely apparent due to intermixed ground vestiture, versus *C. exiguus*: clearly visible as rows of punctures and fine hair like setae devoid of ground vestiture).

**Female.** Length 1.35–1.70 mm (holotype 1.40 mm). Proportions 2.25× as long as wide. Frons simple, convex, flat and shining up to the level of the eyes. Antennal club with three straight or barely procurved sutures marked by a mixture of coarse short setae and longer setae with length approximately the same as distance between sutures. Antennal funiculus with four funicular segments. Pronotal profile widest in line with the centre of the disc. Pronotal margin armed with six serrations, irregularly spaced and sometimes partially fused. Pronotal declivity broadly rounded, with approximately 50 asperities. Pronotal disc occupies approx. one third of the length of the pronotum, gently sloped, uniformly weakly asperate. Pronotal vestiture mostly of golden scale-like setae which are approximately as long as wide, with sparse longer dagger-like setae. Suture between pronotum and elytra almost straight. Scutellum very small, triangular. Elytra 1.8× as long as pronotum, dark brown, broadly rounded with no clear transition to the declivity. Elytral texture rugose, with punctures from the ground vestiture approximately half of the diameter of the strial punctures, and interspaces irregularly rugose. Striae barely apparent, visible as rows of slightly larger punctures and fine, white, hair-like setae. Interstrial bristles erect, weakly flattened with pointed tips, those near the elytral suture are shorter and broader than those in lateral regions. Interstrial ground vestiture tridentate, 1–2× as long as wide, brown, with a weak iridescence, and distributed across the elytra including between strial punctures. Mesocoxae moderately separated, more than distance between metacoxae. Proventriculus not examined.

**Male.** Similar to female except: Length 1.30–1.60 mm. Frons with a smooth transverse carina above the level of the eyes. Pronotal declivity straight, asperities slightly more sparse. Elytra less rugose than female. Protibiae and protarsi with coarse, long setae on the proximal face, near the apex. Last abdominal ventrite clearly emarginated. Proventriculus sutural teeth of irregular size, confused, in two or more rows. Aedeagus very long, weakly sclerotised. Penis apodemes almost as long as penis body. Tegmen with paired penis apodemes approximately as long as they are spaced apart.

###### Recorded plant hosts.

Moraceae: *Morus alba* L., *M. australis* Poir.

###### Distribution.

China (Hebei, Shandong, Shanghai); Taiwan; South Korea.

###### Etymology.

The name is an adjective derived from a combination of the stem of the Latin noun *mōrus* for mulberry, a linking vowel -*i*- and an adjectival suffix *vorus*, meaning eater.

###### Suggested vernacular name.

Chinese: 南桑梢小蠹; English: Southern mulberry bark beetle; Korean: 남방뽕나무애나무좀.

###### Remarks.

This species has widely been misidentified and misspelled as “*Cryphalus exignus*”, with the first instance of the spelling Niisima (1909) referring to Japanese specimens likely of *C. exiguus*. Since the authority is clearly indicated in the earliest instance, we consider this a misspelling of *exiguus*, rather than a *nomen nudum*.

Under the name *C.* “*exignus*”, which probably referred to this species, Yin and Li (1984) present photos of the proventriculus and aedeagus. The proventriculus differs slightly in the width of the patch of apical teeth. No voucher information for these specimens exist, and it is unclear if this variation is from within this new species or included specimens of *C. exiguus*.

Wood and Bright (1992)) list “*Broussonetia kazinoki*, *Celtis sinensis*, *Diospyros kaki*, *Evodia rutaecarpa*, *Ficus* spp., *Morus alba*, *Morus bombysis* [= *australis*] and *Salix* sp.” under *C. exiguus* which may refer to this species. It is also unclear whether these hosts are suitable reproductive hosts.

This species has been widely described as a pest of *Morus*. Named as *exignus*, this species was described to feed on developing buds of mulberry in early spring, sometimes reaching significant numbers to affect yields (Yin et al. 1984).

Using the key by Tsai and Li (1963), specimens of this species would key to *C. manschuricus*, but would differ in the scales on the pronotum, the size, proventriculus and aedeagus.

##### Cryphalus paramangiferae

Taxon classificationAnimaliaColeopteraCurculionidae

Johnson sp. nov.

7C5D35EF-576D-5881-84C4-9EEE523738EE

http://zoobank.org/C5D52474-B833-4564-AE40-FD56BEBA6155

**Figure 15.**
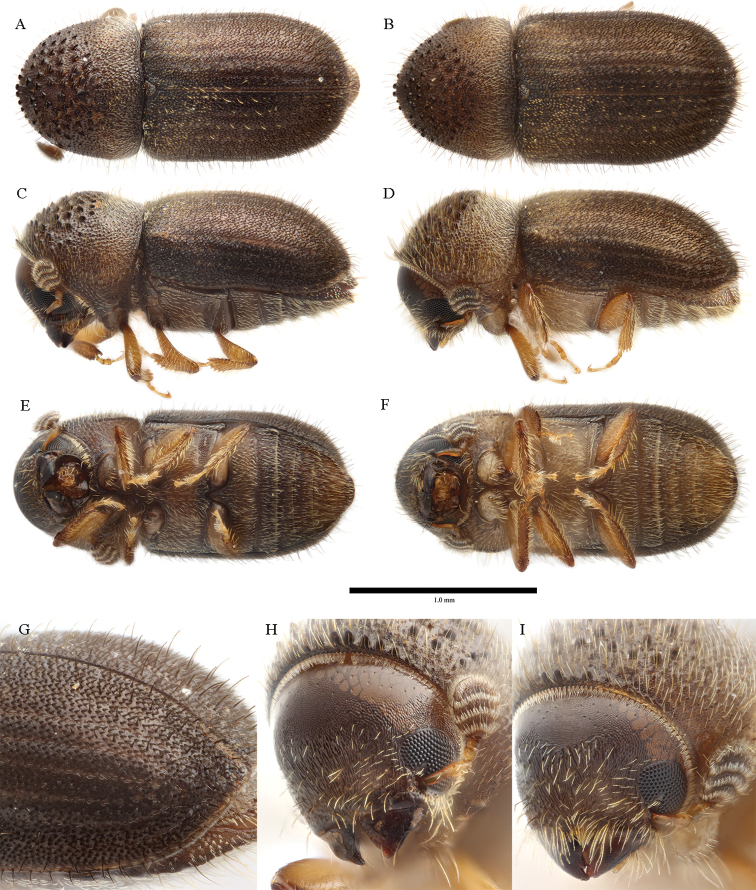
*Cryphalus paramangiferae***A, C, E, G, H** holotype, female, UFFE:34967 **B, D, F, I** paratype, male, UFFE:34969.

Figures 2K , 3K , 15A–I

###### Type material examined.

China • 1 ♀ ***Holotype***; Fujian, Quanzhou, Yongchun, Diyiyan; 25.3169°N, 118.2751°E; 26 Nov. 2015; You Li leg.; ex. *Mangifera indica*; IOZ(E)225765; ex v12347; UFFE:34967; (IOZ) • 1 ♂ ***Paratype***; same collection data; IOZ(E)225766; UFFE:34969; (IOZ) • 1 ♀, 1 ♂ ***Paratypes***; same collection data; pair in gallery with larvae; 1 larva used for DNA extraction without voucher; DNA: 28S:MG051113; UFFE:22108; (UFFE) • 5 ♀♀, 5 ♂♂ ***Paratypes***; same collection data; ex v12352; UFFE:34970; (NHMUK, 1♀, 1♂; FSCA, 1♀, 1♂; MZB, 1♀, 1♂; NIAES, 1♀, 1♂; NMNS, 1♀, 1♂) • 6 ♀♀, 6 ♂♂ ***Paratypes***; Fujian, Quanzhou, Nan’an, Pushan village; 25.1189°N, 118.4276°E; 01 May 2017; You Li leg.; ex. *Mangifera indica*; UFFE:34974; (RIFID, 1♀, 1♂; UFFE, 3♀♀, 3♂♂; USNM, 1♀, 1♂; ZIN, 1♀, 1♂).

###### Other material examined.

China • 1 ♂; Fujian, Quanzhou, Yongchun, Diyiyan; 25.3172°N, 118.2798°E; 17 Nov. 2015; You Li leg.; ex. *Mangifera indica*; UFFE:22062; (UFFE) • 1 ♂; Fujian, Quanzhou, Yongchun, Diyiyan; 25.3169°N, 118.2751°E; 26 Nov. 2015; You Li leg.; ex. *Mangifera indica*; ex v12348; dissected; UFFE:34909 (UFFE) • 1 ♂; same collection data; destructively extracted; DNA: 28S:MG051111; UFFE:25415; (UFFE) • 1 ♂; same collection data; UFFE:34968; (UFFE) • 3; Fujian, Quanzhou, Nan’an, Pushan village; 25.1189°N, 118.4276°E; 28 Apr. 2017; You Li leg.; ex. *Mangifera indica*; UFFE:26956 (UFFE) • 1 ♂ ; same collection data; dissected; mango 5; UFFE:34965 • 2 ♀♀; Guangdong, Shenzhen, Yantian; 22.5889°N, 114.2842°E; 08 Apr. 2017; Wei Lin leg.; EtOH trap; UFFE:33204; (UFFE).

###### Type locality.

China, Fujian, Quanzhou, Yongchun, Diyiyan; (25.317°N, 118.275°E).

###### Diagnosis.

This species is distinguished from other similar *Cryphalus* by the frons with an aciculate texture, the frons of the male which also has a shining patch in the median made from a fine file-like structure, the pronotal disc which is long, and has fine hair-like setae with some bifurcating setae on baso-lateral areas, the elytral striae which are barely impressed, and the striae 1, 2 and 3 which on the declivity, is barely apparent.

This species is externally very similar to *C. mangiferae*. They can be distinguished by the frons of the male (*C. paramangiferae*: with fine shining file like structure versus *C. mangiferae*: identical to female), by a subtle difference in the vestiture of the pronotum (*C. paramangiferae*: very fine and hair like versus *C. mangiferae*: coarse hair like), by a subtle difference in striae 1, 2, and 3 on the declivity (*C. paramangiferae*: slightly impressed and barely apparent with some ground vestiture between punctures versus *C. mangiferae*: apparent without ground vestiture between punctures), by the setae near the dorso-anterior corner of the metaventrite (*C. paramangiferae*: fewer than 10 multifurcate setae not distinct from hair-like setae versus *C. mangiferae*: 20 or more multifurcate setae, all distinctly smaller than other setae), and by the spinulae on the ejaculatory duct inside the aedeagus (*C. paramangiferae*: large spinulae versus *C. mangiferae*: many small spinulae).

**Female.** Length 1.4–1.9 mm (Holotype 1.8 mm). Proportions 2.2× as long as wide. Frons with converging aciculations and weakly emarginated at epistoma. Antennal funiculus usually with 4 segments. Antennal club with three evenly procurved sutures, slightly wider at apex. Pronotal profile widest in line with summit, approx. 0.85× as long as wide. Pronotal margin armed with four to six serrations, the median pair larger and near contiguous. Pronotal declivity with more than 60 asperities (holotype has 68). Pronotal disc approximately one third of the length, gently sloped. Pronotal vestiture entirely fine hair-like, some bifurcating setae bifurcating in baso-lateral area. Suture between pronotum and elytra weakly sinuate. Scutellum shaped as a rounded triangle, almost semi-circular, with sparse, pale, hair-like setae. Elytra 1.6× as long as pronotum, usually slightly darker colour than pronotum, medium-brown, broadly rounded with no clear transition to the declivity. Striae weakly visible as rows of punctures and hair like setae, slightly impressed. Interstrial bristles erect, curving posteriorly, slightly wider at base. Interstrial ground vestiture near triangular, dagger-like, tapering to a fine point, longer on declivity, ground vestiture on basal third are usually a light brown/cold colour. Protibiae and protarsi with only straight, hair-like setae. Mesocoxae moderately separated, much more than metacoxae. Ventrites with mostly hair-like setae. Last abdominal ventrite with margin of rounded tubercles. Proventriculus sutural teeth very weakly sclerotised, barely visible, rounded, in multiple irregular rows each side of suture. Apical teeth in multiple rows, extending almost the width of a segment. Closing teeth long, barely branched, extending beyond masticatory brush with fine, pointed branched tips. Masticatory brush slightly shorter than apical plate.

**Male.** Similar to female except: Length 1.4–1.9 mm. Frons with similar aciculations to females, but upper levels with glabrous patch with extremely fine transverse aciculations. Pronotal profile slightly more triangular, widest nearer base. Pronotal vestiture with fine hair-like setae and some bifurcating setae on baso-lateral areas. Protibiae and protarsi with coarse, curved setae on proximal edge of protibia and on the first few tarsi. Last abdominal ventrite very weakly emarginated. Aedeagus shorter, penis body sclerotised, only slightly tapered to a broadly rounded point at apex, ejaculatory duct with very large, rose-thorn-shaped spinulae, especially along portion inside penis body, end plates large and strongly sclerotised. Penis apodemes 1.5× as long as penis body. Tegmen broad, with very short apodemes.

###### Distribution.

China (Fujian, Guangdong)

###### Etymology.

The name is derived from a combination of the Ancient Greek *παρά*, meaning near, next to or besides, and *mangiferae*, the specific epithet of the species *Cryphalus mangiferae*, a species which is phylogenetically and morphologically very similar. It is invariable.

###### Suggested vernacular name.

Chinese: 伪芒果梢小蠹 ; English: False mango bark beetle.

###### Recorded plant hosts.

Anacardiaceae: *Mangifera indica* L.

###### Remarks.

Johnson et al. (2017) noted a high genetic diversity of *Cryphalus mangiferae* in Asia, suggesting that there are likely cryptic and near-cryptic species present. Two of the specimens used in the molecular study (specimen 54 and 71) are of this newly described species, from the type locality.

The morphological differences are sexual characters, likely to be a reproductive barrier between the two species. In a location near the type locality (Nan’an), a sample reared from one log was of a mixture of *C. mangiferae* and *C. paramangiferae*, though no obvious differences in the biology were noted. All individuals collected in pairs corroborate these differences.

None of the examined types of synonyms of *C. mangiferae* have the fine hair-like setae on the pronotum or less distinct striae.

Based on the key for Chinese *Cryphalus* by Tsai and Li (1963), specimens of this species would fail on couplet 2/3, because the male frons does not have a prominent carina and the spinulae on the ejaculatory duct are distinctly spine-like rather than setiform.

##### Cryphalus scopiger

Taxon classificationAnimaliaColeopteraCurculionidae

Berger, 1917

726AE100-4B43-527B-B22E-8FC2B4FC79FC

Fig. 16 A–D

Cryphalus scopiger Berger, 1917: 228 (Russia).

###### Type material examined.

Russia • 1 ♂ ***Lectotype***; “Ю.-Уссур. Край, окр. г. Владивост., ст. Седанка” [Southern Ussuriyskiy Kray, now Primorskiy Kray, near Vladivostok city, Sedanka station]; 1915; “В. Бергеръ” [V. M. Berger leg.]; ex. *Juglans mandshurica*; UFFE:34758 (ZIN).

###### Other material examined.

China • 1 ♀; 辽宁清源N5 [Liaoning, Qingyuan N5]; 01 May 1987; 宋友文 [Youwen Song leg.]; 核桃楸 [*Juglans mandshurica*]; IOZ(E)700992; UFFE:33471; (IOZ) • 1 ♂; 辽宁清源N5 [Liaoning, Qingyuan N5]; 01 May 1987; 宋友文 [Youwen Song leg.]; 核桃楸 [*Juglans mandshurica*]; IOZ(E)700978; UFFE:33472; (IOZ).

Russia • 2 ♀♀; “228. Майх. Оп. Л.” [Primorskiy Kray, Shkotovskiy District, Maikhe educational and experimental forest]; “усох ветки *Jugl. mand.*” [dry limbs of *Juglans mandshurica*]; label in A.V. Mishin’s handwriting; UFFE:34763; (ZIN) • 1 ♀ “дол. р. Майхэ, Шкотовск. р. ДВК [Far Eastern Kray, now Primorskiy Kray, Shkotovskiy District, Maikhe River valley]; “V. 931” [May 1931]; “Куренцов” [A.I. Kurentsov leg,]; UFFE:34760; (ZIN) • 1 ♂; “248”. [Primorskiy Kray, Shkotovskiy District, Maikhe educational and experimental forest]; “21.VIII.31” [21 Aug 1931]; “A.M.” [A.V.Mishin leg.]; ex. *Juglans mandshurica*; Specific locality data not provided, interpreted from specimens with otherwise similar collection data; UFFE:34764; (ZIN) • 1 ♀; same collection data except “28.VIII.31” [28 Aug 1931]; UFFE:34765; (ZIN) • 1 ♀; “р. Майхэ, лесн. Шкотовск. р. ДВК” [Far Eastern Kray, now Primorskiy Kray, Shkotovskiy District, Maikhe educational and experimental forest, Maikhe river]; “28./VIII 931” [28 Aug 1931]; “Шаблиовский и Любарский” [V. V. Shabliovskiy, and L. V. Lyubarskiy leg.]; UFFE:34762; (ZIN)• 1 ♀; Primorskiy Kray, Shkotovskiy District, Forest district No. 11; 11 Sep. 1931; A. V. Mishin leg.; ex. *Juglans mandshurica*; no.169; UFFE:31840; (FSCA) • 1 ♀; same collection data; UFFE:31841; (FSCA) • 3 ♀♀, 4 ♂♂; “дол. р. Майхэ, Шкотовск. р. ДВК” [Far Eastern Kray, now Primorskiy Kray, Shkotovskiy District, Maikhe river valley]; “V.932” [May 1932]; “Любарский” [L.V. Lyubarskiy leg.]; “из коллекции В.Н. Старка” [from V.N. Stark collection]; UFFE:34761; (ZIN) • 1 ♀, 1 ♂; “ дол. р. Малазы, Сучан. р., Усс. кр.” [Ussuriyskiy Kray, now Primorskiy Kray, Suchan District, Malasa River valley]; “V.931” [May 1931]; “Куренцов” [A. I. Kurentsov leg.]; “32a// из коллекции В.Н. Старка” [from V.N. Stark collection]; UFFE:34759; (ZIN).

###### Diagnosis.

This species can be distinguished from others in East Asia by the size (usually 1.70–2.0 mm), by the weakly aciculate frons without apparent sexual dimorphism, by the antennae with nearly straight sutures, by the pronotal disc occupying one third of the pronotal length, with entirely hair like setae, and by the interstrial ground vestiture of the female at the apex of the declivity, which is dense and elongate forming a brush.

**Female.** Length 1.70–2.05 mm. Proportions 2.23× as long as wide. Frons simple, convex, in the lower portion with aciculations converging at epistoma, and with indistinct longitudinal median keel, in the upper portion with sparsely set punctures, faintly shining, covered by golden hairs, longer above epistoma. Antennal club with three nearly straight sutures marked by setae (fourth suture only indistinctly feebly marked) at outer surface and three strongly procurved sutures at inner surface. Antennal funiculus with four funicular segments, the pedicel is shorter than the other segments combined. Pronotal colour brown, similar to head and elytra (may be most beetles are incompletely coloured since the original description (Berger 1917) gives mature beetles colour as black). Pronotal profile 0.82 as long as wide, slightly triangular, widest in posterior third, distinctly narrowing towards the base, rather strongly narrowing anteriorly, posterior angles strongly rounded. Pronotal margin rounded, armed with six serrations at the anterior margin, central denticles larger than lateral. Pronotal declivity with approximately 50 asperities. Pronotal disc approximately one third of the pronotal length, sloping rather strongly from the summit. Pronotal vestiture long, golden and hair-like on disc, with dense, long hairs on the postero-lateral margin. Suture between pronotum and elytra weakly sinuate, base of the pronotum is marked with fine carina. Scutellum V-shaped. Elytra 1.47× as long as wide, 1.9× as long as pronotum, 1.05× wider than pronotum, parallel-sided for more than 3/4 of length, then broadly rounded toward apex. Striae impressed, interstriae convex, minutely punctured; punctures in striae round, with flat bottom, densely set, but not fusing with neighbouring punctures. Interstrial bristles erect, hair-like with pointed apices. Interstrial ground vestiture scale-like, formed by elongated pointed scales 2.0× as long as wide arranged in four rows at each interstria. Apex of elytra obtuse, slightly projecting, with dense brush of short hair-like brown setae. Protibiae and protarsi with only straight, hair-like setae. Mesocoxae moderately separated, a little more than distance between metacoxae. Proventriculus (according to Berger 1917, sex of specimen dissected not indicated) typical of the genus. Proventriculus sutural teeth in a narrow band, rather numerous, blunt and rounded tubercles arranged in two irregular rows along each side of the suture. Apical teeth arranged in four rows, central teeth long, faintly curved, lateral teeth much smaller in size and disappear beyond the centre of each half of apical plate, not extending up to its lateral margins. Closing teeth usually in number of ten deeply palmate, longer than masticatory brushes. Masticatory brush as long as apical plate.

**Male.** Similar to female except: Length 1.70–1.95 mm (lectotype 1.75 mm). Elytra slightly stouter than in the female, about 1.39–1.43 as long as wide. Apex of elytra with hair-like setae similar to rest of declivity. Aedeagus (according to Berger 1917) long, penis body with developed end plates. Penis apodemes 1.5× as long as penis body. Tegmen with short paired tegminal apodemes.

###### Recorded plant hosts.

Juglandaceae: *Juglans mandshurica* Maxim.; Oleaceae: *Fraxinus mandshurica* Rupr. (Krivolutskaya 1996)

###### Distribution.

Russia (Primorskiy Kray); North Korea (Ju 1964); China (Liaoning).

###### Suggested vernacular name.

Russian: Ореховый крифал; Chinese: 胡桃楸梢小蠹

###### Remarks.

*Cryphalus scopiger* is a very common species that builds galleries in thin bark of stems and drying limbs of Manchurian walnut in river valleys. Wu et al. 1991 reported *Cryphalus viburni* as a pest of *Juglans mandshurica* in northern China, with detailed descriptions of the phenology and excavation of non-reproductive galleries. We found specimens deposited in IOZ with the corresponding collection information (i.e., implicit vouchers) labelled as *C. viburni*, which were determined to be *C. scopiger*. This is corroborated with the host plant information. The descriptions provided were very similar in wording and content to those by Yin et al. (1984), and did not match the specimens vouchered, presumably not based on directly examined material. Song et al. (1998) also reports the same species on *Prunus*, though specimens were not verified.

The size range has been described as 1.50–1.90 (original description, Berger 1917) and 1.50–2.00 (Krivolutskaya 1996). The smallest examined was 1.7 mm, based on the material examined listed above and 200 additional specimens deposited at ZIN.

**Figure 16.**
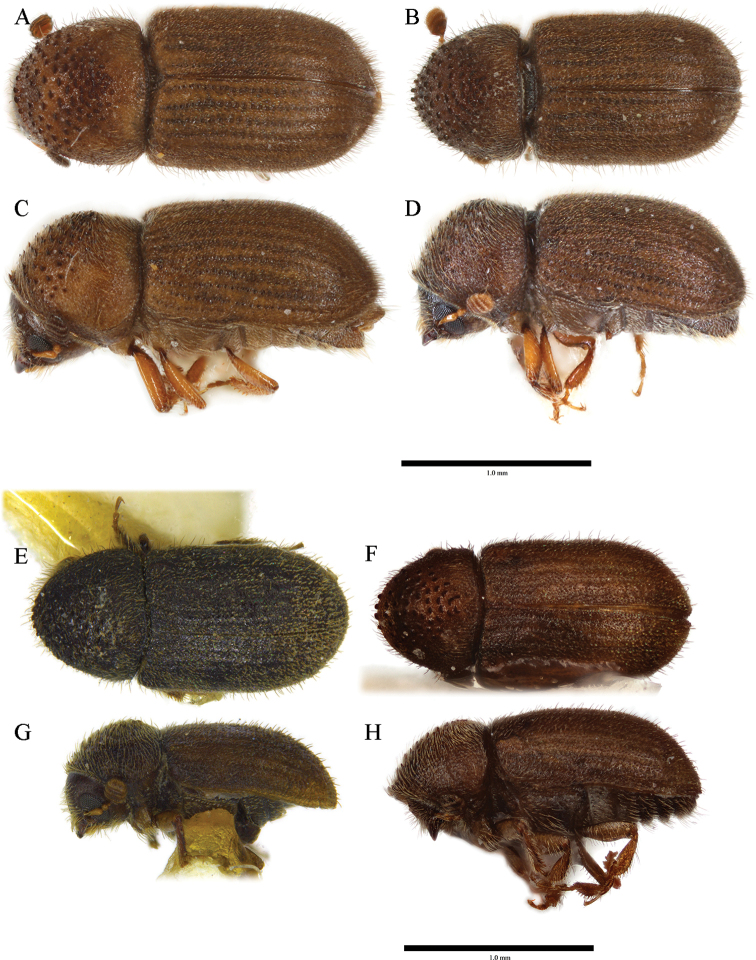
**A–D***Cryphalus scopiger***A, C** female, UFFE:33471 **B, D** male, UFFE:33472 **E–H***Cryphalus viburni***E, G** paralectotypes (2 different specimens, sex unknown), UFFE:34767 **F, H** male, UFFE:31842 [**H** is flipped horizontally].

##### Cryphalus viburni

Taxon classificationAnimaliaColeopteraCurculionidae

Stark, 1936

B89BC5DA-4B2F-5AE9-B7EA-C4DCCA3930F8

Figure 16E–H

Cryphalus viburni Stark, 1936: 151 (Russia) Eggers, 1942: 30 (Russia).

###### Type material examined.

Russia • 1 ***Lectotype***; “Шкотово” [Primorskiy Kray, Shkotovo]; “VI.1929” [Jun.1929], “Шаблиовск.” [V.V. Shabliovskiy leg.]; “*Cryphalus viburni* Stark. Typ. V. Stark det. 1932 //Lectotypus *Cryphalus viburni* Stark Michalski J. 1965 [des.]”; UFFE:34766 (ZIN) • 7 ***Paralectotypes***; same collection information except labelled with “*Cryphalus viburni* Stark. Typ. V.Stark det. 1932// Paralectotypus”; UFFE:34767 (ZIN).

###### Other material examined.

Russia • 1 ♀; Primorskiy Kray, Laso Nature Reserve, Sukhoy River post; 15 Aug. 1990; M. Yu. Mandelshtam leg.; under bark of *Viburnum sargentii*; UFFE:31842 (FSCA) • 9 ♀♀, 3 ♂♂; same collection data; UFFE:34768 (ZIN).

###### Diagnosis.

*Cryphalus viburni* can be identified by the size (1.40–1.70 mm), by the aciculate frons, by the antennal club with slightly recurved, almost straight sutures, by the short pronotal disc (1/4 of the length), by the entirely hair-like setae on the pronotal disc, by the hair-like interstrial bristles of approximately equal length over the elytra.

*Cryphalus viburni* can be distinguished from similar *Cryphalus scopiger* by the smaller size (*C. viburni*: 1.40–1.70 mm, versus *C. scopiger*: 1.70–1.95 mm), by the profile of the pronotum (*C. viburni*: more semi-circular, widest at base, versus *C. scopiger*: widest in line with summit), by the elytra sculpturing (*C. viburni*: striae barely impressed, versus *C. scopiger*: striae impressed), and by the interstrial ground vesiture at elytral apex of the females (*C. viburni*: similar to rest of elytra, versus *C. scopiger*: dense and elongated making a brush).

*Cryphalus mangiferae* and *C. paramangiferae* are somewhat similar but can be easily distinguished by the antennal sutures (*C. viburni*: almost straight, versus *C. mangiferae* and *C. paramangiferae*: procurved), and by the profile of the pronotum and the size of the pronotal disc (*C. viburni*: disc one quarter of pronotal length, pronotum widest at base, versus *C. mangiferae* and *C. paramangiferae*: disc one third of pronotal length, widest near in line of summit).

**Female.** Length 1.50–1.70 mm. Proportions variable, 2.06–2.36 as long as wide. Frons simple, convex, in the lower portion with aciculations converging at epistoma, and fine median keel, sometimes obscure, in the upper portion with sparsely set punctures, faintly shining. Antennal club with three slightly recurved, nearly straight sutures marked by setae (fourth suture only indistinctly feebly marked) at outer surface and three strongly procurved sutures at inner surface. Antennal funiculus with four funicular segments, total length short, less than half of length of club. Pronotal colour black, similar to head and elytra. Pronotal profile transverse (and not longitudinal or of equal length and width as stated by Krivolutskaya, 1996), 0.75–0.86 as long as wide, semi-circular, widest at base, with parallel sides in posterior third and rather broadly rounded anteriorly, posterior angles nearly rectangular. Pronotal margin rounded, armed with four to six serrations at the anterior margin, rather widely spaced, central denticles larger than lateral. Pronotal declivity with approximately 50 asperities. Pronotal disc approximately one fourth of the pronotal length, sloping rather strongly from the summit. Pronotal vestiture short, hair-like, golden, on disc (without scale-like setae). Suture between pronotum and elytra sinuate, base of the pronotum is marked with fine carina. Scutellum V-shaped. Elytra variable in proportions, 1.43–1.57× as long as wide, 2.0–2.26× as long as pronotum, 1.07× wider than pronotum, parallel-sided for 3/4 of length, then broadly rounded toward apex. Striae not strongly impressed, interstriae only slightly convex, punctures in striae without flat bottom, touching neighbouring punctures, elytral surface only faintly shining. Interstrial bristles erect, hair-like, of even moderate length along elytra, with pointed apices. Interstrial ground vestiture scale-like arranged in three-four rows at each interstria consists of rather short blunt scales 1.5–2.0× as long as wide. Protibiae and protarsi with only straight, hair-like setae. Mesocoxae separated barely more than metacoxae. Proventriculus not examined.

###### Male.

Length 1.45–1.70 mm. Similar to female, except elytra slightly stouter than in female. Aedeagus not studied. Proventriculus not studied.

###### Recorded plant hosts.

Adoxaceae: *Viburnum sargentii* Koehne, *V. dilatatum* Thunb. (Yin et al. 1984 [unconfirmed]).

###### Distribution.

Russia (Primorskiy Kray); China (Shaanxi (Tsai and Li 1963 [unconfirmed]); Shanxi (Alonso-Zarazaga et al. 2017 [unconfirmed])).

###### Suggested vernacular name.

Russian: Калиновый крифал; Chinese: 荚蒾梢小蠹.

###### Remarks.

Despite discovering that this species had been incorrectly reported from northern China, we expect it to be present. We were unable to corroborate records from Shaanxi, or records collected on *Viburnum dilatatum* as a host, initially mentioned by Yin et al. (1984) without citing any collections.

## Conclusions

*Cryphalus* is remarkably diverse in East Asia, but most available literature does not allow for accurate species identification, particularly for taxa in tropical and subtropical broadleaf forests. This work changes number of valid species of *Cryphalus* to 254, based on the taxa listed in Johnson et al. (2020); four additional taxa described here, and two taxa now recognized as synonyms. Thorough descriptions, redescriptions, photographs, and sequence data enable accurate and precise identification and further study of these minute bark beetles.

## Supplementary Material

XML Treatment for Cryphalus

XML Treatment for Cryphalus artocarpus

XML Treatment for Cryphalus dilutus

XML Treatment for Cryphalus dorsalis

XML Treatment for Cryphalus exiguus

XML Treatment for Cryphalus gnetivorus

XML Treatment for Cryphalus itinerans

XML Treatment for Cryphalus kyotoensis

XML Treatment for Cryphalus lipingensis

XML Treatment for Cryphalus mangiferae

XML Treatment for Cryphalus meridionalis

XML Treatment for Cryphalus morivorus

XML Treatment for Cryphalus paramangiferae

XML Treatment for Cryphalus scopiger

XML Treatment for Cryphalus viburni

## Supplementary materials

Supplementary material 1 Summary of sequences used for the phylogeny of *Cryphalus* spp. Data type molecular data https://binary.pensoft.net/file/476790This dataset is made available under the Open Database License (http://opendatacommons.org/licenses/odbl/1.0/). The Open Database License (ODbL) is a license agreement intended to allow users to freely share, modify, and use this Dataset while maintaining this same freedom for others, provided that the original source and author(s) are credited.Andrew J. Johnson
